# *Aethionema arabicum* genome annotation using PacBio full‐length transcripts provides a valuable resource for seed dormancy and Brassicaceae evolution research

**DOI:** 10.1111/tpj.15161

**Published:** 2021-02-08

**Authors:** Noe Fernandez‐Pozo, Timo Metz, Jake O. Chandler, Lydia Gramzow, Zsuzsanna Mérai, Florian Maumus, Ortrun Mittelsten Scheid, Günter Theißen, M. Eric Schranz, Gerhard Leubner‐Metzger, Stefan A. Rensing

**Affiliations:** ^1^ Plant Cell Biology Department of Biology University of Marburg Marburg Germany; ^2^ School of Biological Sciences Royal Holloway University of London Egham Surrey UK; ^3^ Matthias Schleiden Institute/Genetics Friedrich Schiller University Jena Jena Germany; ^4^ Gregor Mendel Institute of Molecular Plant Biology Austrian Academy of Sciences Vienna BioCenter (VBC) Vienna Austria; ^5^ Université Paris‐Saclay INRAE URGI Versailles 78026 France; ^6^ Biosystematics Group Wageningen University Wageningen The Netherlands; ^7^ Laboratory of Growth Regulators Centre of the Region Haná for Biotechnological and Agricultural Research Palacký University and Institute of Experimental Botany Academy of Sciences of the Czech Republic Olomouc Czech Republic; ^8^ BIOSS Centre for Biological Signaling Studies University of Freiburg Freiburg Germany; ^9^ LOEWE Center for Synthetic Microbiology (SYNMIKRO) University of Marburg Marburg Germany

**Keywords:** *Aethionema arabicum*, genome annotation, seed germination, Brassicaceae evolution, alternative splicing, transcription factors, Iso‐seq

## Abstract

*Aethionema arabicum* is an important model plant for Brassicaceae trait evolution, particularly of seed (development, regulation, germination, dormancy) and fruit (development, dehiscence mechanisms) characters. Its genome assembly was recently improved but the gene annotation was not updated. Here, we improved the *Ae. arabicum* gene annotation using 294 RNA‐seq libraries and 136 307 full‐length PacBio Iso‐seq transcripts, increasing BUSCO completeness by 11.6% and featuring 5606 additional genes. Analysis of orthologs showed a lower number of genes in *Ae. arabicum* than in other Brassicaceae, which could be partially explained by loss of homeologs derived from the At‐α polyploidization event and by a lower occurrence of tandem duplications after divergence of Aethionema from the other Brassicaceae. Benchmarking of MADS‐box genes identified orthologs of *FUL* and *AGL79* not found in previous versions. Analysis of full‐length transcripts related to ABA‐mediated seed dormancy discovered a conserved isoform of *PIF6‐β* and antisense transcripts in *ABI3*, *ABI4* and *DOG1*, among other cases found of different alternative splicing between Turkey and Cyprus ecotypes. The presented data allow alternative splicing mining and proposition of numerous hypotheses to research evolution and functional genomics. Annotation data and sequences are available at the *Ae*. arabicum DB (https://plantcode.online.uni‐marburg.de/aetar_db).

## Significance Statement

Improved gene annotation of *Aethionema arabicum* using long‐read transcript sequencing provides a plethora of full‐length isoforms and an important resource for Brassicaceae evolution studies.

## INTRODUCTION

The Brassicaceae species *Aethionema arabicum* has become an important model system for unraveling the control and development of seed and fruit traits because it displays an unusual phenomenon, diaspore (fruit/seed) heteromorphism. It produces two types of fruits, a bigger and dehiscent one that produces two to six mucilaginous seeds (M+) and an indehiscent one, which only contains one non‐mucilaginous seed (M‐) (Lenser *et al*., 2016). This allows alternative strategies for dispersion of the seeds and timing of germination depending on the environmental conditions. M+ seeds may stick to the substrate and remain close to the mother plant, while M‐ seeds, inside an indehiscent fruit with wings, may be dispersed across longer distances by wind or water currents (Arshad *et al*., 2019). Moreover, *Ae. arabicum* shows plasticity for the proportion of the two fruit types depending on environmental responses such as temperature and nutritional or herbivory stress (Lenser *et al*., 2016; Bhattacharya *et al*., 2019a, 2019b,2019a, 2019b). Therefore, *Ae. arabicum* is an excellent model to study seed development, regulation, germination, dormancy and fruit dehiscence mechanisms (Mohammadin *et al*., 2017; Lenser *et al*., 2018; Arshad *et al*., 2019; Mérai *et al*., 2019; Bhattacharya *et al*., 2019b). Additionally, multiple resources and studies are available for two ecotypes of *Ae. arabicum*, from Turkey (TUR) or Cyprus (CYP) (Mohammadin *et al*., 2018; Mérai *et al*., 2019; Nguyen *et al*., 2019), adapted to different environmental conditions (Bhattacharya *et al*., 2019b) and differently reacting to stresses and environmental stimuli, such as in response to light during germination (Mérai *et al*., 2019).

*Aethionema arabicum* is a member of the tribe Aethionema, sister group to all other ‘crown group’ Brassicaceae species. The crown group includes many plants of agricultural interest such as cabbage (*Brassica oleracea*), rapeseed (*Brassica napus*) and mustard (*Brassica rapa*), as well as the model plant *Arabidopsis thaliana* (Nikolov *et al*., 2019). Brassicaceae species, including *Ae. arabicum*, went through five ancient polyploidization events in their evolutionary history: near the origin of seed plants (At‐ζ), in flowering plants (At‐ε), in eudicots (ancient hexaploidy, At‐γ) and in part of the Brassicales including the sister group of Brassicaceae (Cleomaceae) (At‐β), and importantly, all Brassicaceae species including *Ae. arabicum* share the At‐α whole genome duplication (WGD) (Schranz *et al*., 2012; Cheng *et al*., 2013; Walden *et al*., 2020). Due to its phylogenetically important position and shared WGD history, *Ae. arabicum* greatly facilitates evolutionary, comparative genomic and gene family phylogenetic analyses to understand genome and trait evolution of crucifers (Schranz *et al*., 2012; Nguyen *et al*., 2019; Walden *et al*., 2020). Additionally, Brassicaceae produce glucosinolates, secondary metabolites involved in plant defense against biotic and abiotic stresses (Del Carmen Martinez‐Ballesta *et al*., 2013; Bhattacharya *et al*., 2019a), which is another interesting trait also present in *Ae. arabicum* (Mohammadin *et al*., 2017; Mohammadin *et al*., 2018; Bhattacharya *et al*., 2019a).

Genome annotation can be addressed using different strategies, with increasing accuracy depending on the time and effort invested and the quality of the evidence supporting gene structure prediction (Yandell and Ence, 2012). The simplest method would be based only on *ab initio* predictors and the most complex one would use annotation pipelines based on transcript and protein evidence, followed by optional manual curation using a genome browser to polish the results that automatic annotation cannot resolve (Yandell and Ence, 2012). Alternative methods are sometimes used to migrate previous gene versions to new genome versions using lift‐over tools (Keilwagen *et al*., 2016; Pracana *et al*., 2017). In most cases, genome annotation is based on short‐read assemblies reconstructed using transcript assemblers (Grabherr *et al*., 2011; Pertea *et al*., 2015). However, most of the multi‐exon genes have been observed to be alternatively spliced in plants and animals (Zhang *et al*., 2017; Hardwick *et al*., 2019), and often short reads cannot cover the full length of transcripts, which makes it hard to reconstruct the correct combination of exons of alternative isoforms *in silico* (Hardwick *et al*., 2019). Alternative splicing (AS) produces multiple transcripts that may yield different proteins from the same gene. Additionally, AS can lead to different untranslated regions (UTRs), which might be important for post‐transcriptional regulation and may affect mRNA transport, stability, translation efficiency and subcellular localization (Grillo *et al*., 2010; Kelemen *et al*., 2013). Interestingly, it has been observed that AS might play an important role in seed germination (Zhang *et al*., 2016; Narsai *et al*., 2017), involving key genes such as *DELAY OF GERMINATION 1* (*DOG1*), which affects seed dormancy (Nakabayashi *et al*., 2015).

Recently, version 3 (V3) of the *Ae. arabicum* genome of the TUR ecotype was assembled and organized in 11 linkage groups (based on a linkage map derived from crossing the TUR ecotype with the CYP ecotype) and 2872 unordered scaffolds (Nguyen *et al*., 2019), with 65.5% of the genome sequence covered by the linkage groups. This genome version did not predict gene models *de novo*, but lifted over the gene models from v2.5, which were lifted before from v1.0. This process caused formatting inconsistencies and gene structure annotation errors such as incorrect stop codons, incomplete genes, missing features and frameshifts, which were carried over from annotation v1.0 through v2.5 to v3.0. Here, we present the gene model annotation v3.1 for *Ae. arabicum*, based on the genome assembly V3 and predicting the gene model structure *de novo*, using MAKER (Campbell *et al*., 2014). In total, 294 Illumina RNA‐seq samples from multiple tissues and PacBio full‐length transcript sequences of seeds and leaves of the ecotypes TUR and CYP were used, capturing full‐length isoform sequences and UTRs that were used to improve the genome annotation of this model plant.

## RESULTS AND DISCUSSION

### Annotation workflow overview

**Figure 1:**
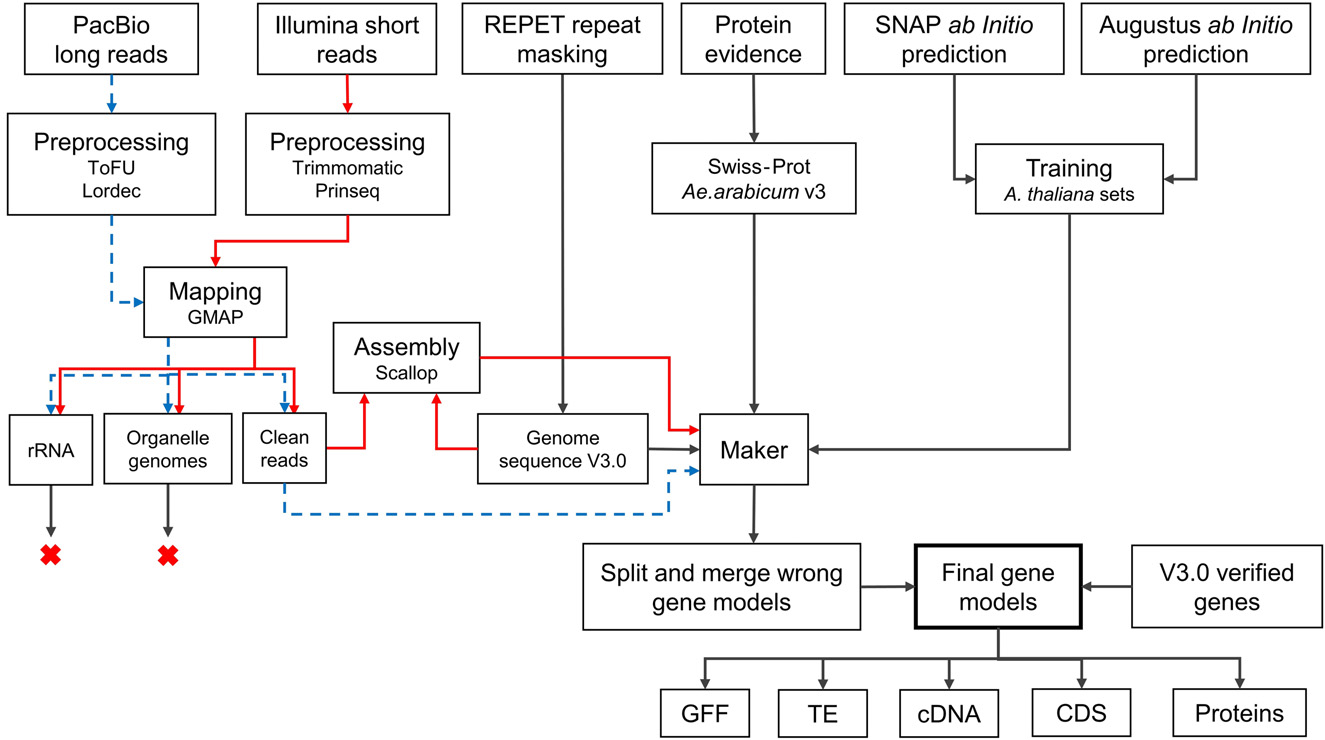
Gene annotation workflow based on MAKER and supported by evidence from multiple sources. Transcripts from long and short reads were used as evidence for gene expression. Swiss‐Prot Embryophyta proteins and predicted proteins free of errors from gene annotation v3.0 were used to support the gene prediction. SNAP and Augustus were used as *ab initio* predictors. Red arrows show analysis steps for short reads, blue dashed arrows for long reads. Red X’s represent discarded reads because they were mapped to organelle or rRNA sequences.

*Aethionema Arabicum* genome assembly versions are described using capital version letters (V), annotation versions are described using lower‐case v. To generate the gene annotation v3.1, the *Ae. arabicum* genome sequence V3 was used as reference, i.e., the same genome version as in gene annotation v3.0 (Nguyen *et al*., 2019). Repeat masking was carried out using REPET (Quesneville *et al*., 2005), and SNAP (Korf, 2004) and Augustus (Stanke *et al*., 2006) were used as *ab initio* gene predictors. Transcriptome sequences based on short and long reads were used as evidence of gene expression, and proteins of Embryophyta (land plants) from Swiss‐Prot (UniProt, 2019) and verified gene models from *Ae. arabicum* v3.0 free of evident errors (incorrect stop codons, incomplete genes, missing features, frameshifts and nucleotide sequences not multiples of three) were used to support the gene prediction (Figure 1).

After processing reads and discarding those mapping to rDNA or organelles, clean short reads were assembled using Scallop (Shao and Kingsford, 2017) with the genome‐guided method. Cleaned PacBio full‐length reads together with the transcripts produced by Scallop and the protein evidences were provided to MAKER (Campbell *et al*., 2014). Subsequently, the output of MAKER was improved by fixing incorrectly merged or split gene models and by adding well‐supported gene models from v3.0 missing in MAKER results. The final genes were formatted to produce the general feature format (GFF) and sequence files (Figure 1). Details of the steps of the pipeline can be found in the next subsections and in the Experimental Procedures.

### Repeat content

**Figure 2:**
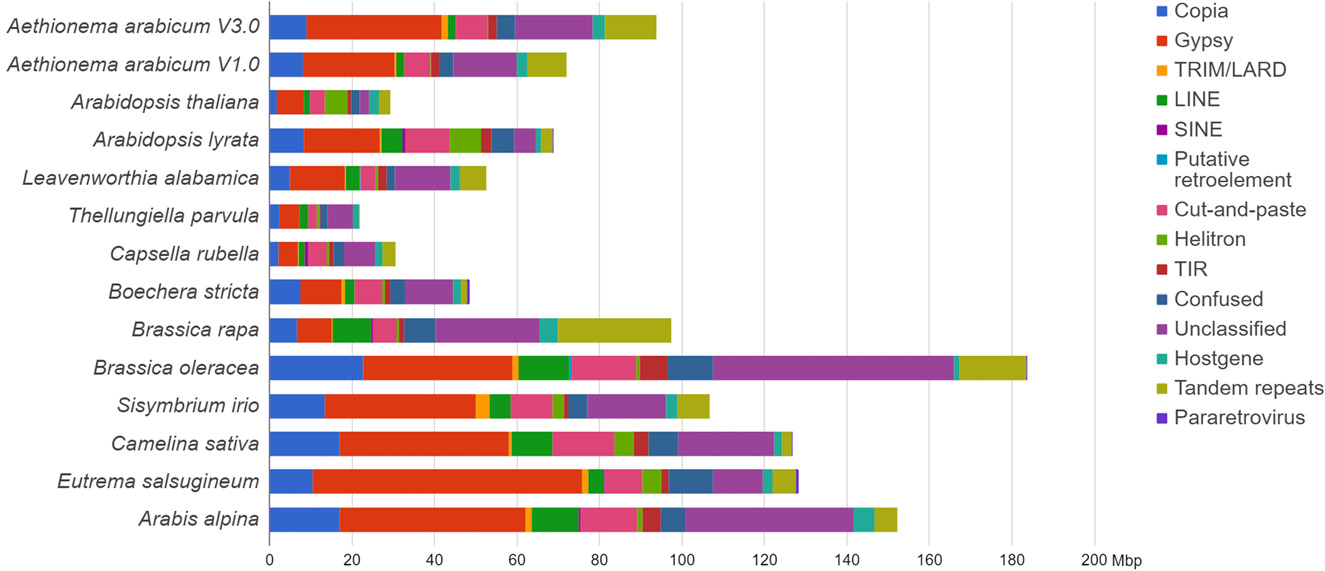
Comparison of repetitive element complements across Brassicaceae. All genome assemblies were annotated using the REPET approach described in the Experimental Procedures. Bars indicate the cumulative coverage in Mbp of different classes of repeats for each genome assembly.

We performed *de novo* prediction and annotation of repetitive elements in the genome V3.0 assembly using REPET. Repetitive elements were found to contribute 93.4 Mbp (45.9%) of the assembly and 49.5% of the non‐gap genome sequence, i.e., twice the fraction found in *A. thaliana* using a similar approach (Maumus and Quesneville, 2014). Repeat annotation in V3.0 presents a striking increase compared to the 72 Mbp (42.3% of the nucleotide sequence) in the initial public assembly V1.0 (Haudry *et al*., 2013). We compared the contribution of different classes of repetitive elements in *Ae. arabicum* V3.0 to that found in 12 other Brassicaceae genomes (Figure 2). As in several Brassicaceae assemblies, the repeat content is dominated by Gypsy‐type long terminal repeat (LTR) retrotransposons which compose 32.7 Mbp of the assembly and almost 35% of the repeat complement. Accordingly, almost half of the difference in repeat content compared to the initial assembly is attributed to Gypsy‐type elements, which is consistent with significantly improved incorporation of heterochromatic regions in the V3.0 assembly (Nguyen *et al*., 2019). We also observed that, although the completeness of the genomes compared here is variable, the repetitive element fraction detected in the *Ae. arabicum* V3.0 assembly is in the upper range of that found in Brassicaceae genomes of similar size (Figure S1).

### Long‐read transcriptome

A total of 418 880 PacBio reads of insert (ROIs) from a pool of total RNA from seeds and leaves of two *Ae. arabicum* ecotypes (210 667 ROIs from CYP and 208 213 ROIs from TUR) were generated. Total RNA of each ecotype was split by fragment length in three parts for Iso‐seq, using 15.74 sequencing passes on average, yielding transcripts with 1533 bp length and 95.3% quality on average (Table S1). The Transcript Isoforms Full length and Unassembled (ToFU) pipeline (Gordon *et al*., 2015) was used to identify PacBio full‐length transcripts. In total, 207 577 reads were classified as full‐length non‐chimeric reads; 101 721 from the CYP ecotype and 105 856 from the TUR ecotype. After using ToFU’s clustering, 67 312 CYP full‐length consensus sequences and 72 095 TUR full‐length consensus sequences were obtained. In total, for both ecotypes there were 139 407 full‐length consensus sequences, which were derived from the initial 418 880 ROIs. After discarding sequences mapping to rDNA and organelles, a total of 136 307 sequences were mapped to the *Ae. arabicum* V3 genome.

### Testing several assemblers for short‐read gene expression evidence

MAKER requires assembled transcripts as expression evidence input. A total of 294 RNA‐seq samples sequenced with Illumina technology were used to test assembled transcriptomes fed to MAKER. Most of them were very short single‐end (SE) reads (50 bp) with a minimum length of 30 bp after pre‐processing. Hence, three short‐read assemblers were tested using their genome‐guided mode: Trinity (Grabherr *et al*., 2011), Scallop (Shao and Kingsford, 2017) and StringTie (Pertea *et al*., 2015). Additionally, PASA (Haas *et al*., 2003), known to be able to create comprehensive transcriptome assemblies combining transcripts from several sources (Wang *et al*., 2016), was used to integrate the StringTie assembly together with the PacBio full‐length transcripts. All the MAKER tests with short‐read assemblers provided the PacBio transcripts separately, whereas PASA included them already in a comprehensive assembly.

The results of MAKER using one or another of these assemblers were similar. They yielded between 24 224 and 24 761 genes and comparable results with regard to the number of genes supported by PacBio full‐length transcripts, BUSCO completeness (Simao *et al*., 2015), Gene Ontology (GO) terms and protein domain evidence (Table 1). Manual inspection of the results in JBrowse (Buels *et al*., 2016) showed that using any of the assemblers as input for MAKER produced similar results. Yet, none of the gene model sets produced were perfect. For the inspected cases, 100 random genes were checked to see if gene models were consistent with evidences from protein sequences and PacBio full‐length transcripts. MAKER results did not indicate superior performance of any of the different assemblers. The Scallop transcriptome representation was selected as the input for the final annotation in MAKER because it showed the highest number of genes supported by PacBio full‐length transcripts, the highest number of protein domains associated and the second highest BUSCO completeness and number of GO terms. The BUSCO completeness for the output of MAKER using Trinity was 0.1% higher, but it showed worse results for all the other parameters tested. StringTie produced very similar results to Scallop (Table 1).

**Table 1:** Differences in MAKER results based on the transcriptome assemblers used

	Scallop	StringTie	Trinity	PASA^c^
Transcript number^a^	24 647	24 761	24 607	24 224
Supported by full‐length transcripts	**15** **665**	15 663	15 504	15 232
BUSCO completeness	94.6%	94.3%	**94.7%**	93.9%
GO terms associated	32 452	**32** **478**	32 410	32 388
Protein domains associated	**34** **667**	34 665	34 657	34 519
Gene average length (bp)	2614	2654	2581	2619
Transcriptome contigs^b^	368 976	68 932	259 665	52 894
Contig average length	701	1185	762	1640

The highest value per row is shown in bold face.

^a^ The top rows of the table include information of the gene models generated by MAKER using as gene expression evidence the transcriptomes assembled by the different programs.

^b^ The bottom rows include data for the transcriptomes generated by Scallop, StringTie, Trinity or PASA.

^c^ PASA assembly includes PacBio full‐length as well as the StringTie transcripts. The other assemblies include only short read‐based transcriptomes. In these cases, the PacBio full‐length transcripts were added as a separate input for MAKER.

PASA generated a more comprehensive transcriptome *per se* than the short‐read assemblers (Table 1, bottom rows), featuring the smallest number of transcripts (52 894) and the longest transcripts on average (1640 bp). However, the most fragmented transcriptome, generated by Scallop (368 976 contigs), produced better results in MAKER than the one combining StringTie and the PacBio full‐length transcripts using PASA. The results of MAKER using PASA transcripts as input showed the lowest values in all the metrics checked (Table 1). MAKER with Scallop produced a 0.7% higher BUSCO completeness and 433 more genes supported by full‐length transcripts than PASA. The average gene length of MAKER output using Scallop or PASA was very similar: 2614 and 2619 bp, respectively (Table 1).

### Split and merged genes

**Figure 3:**
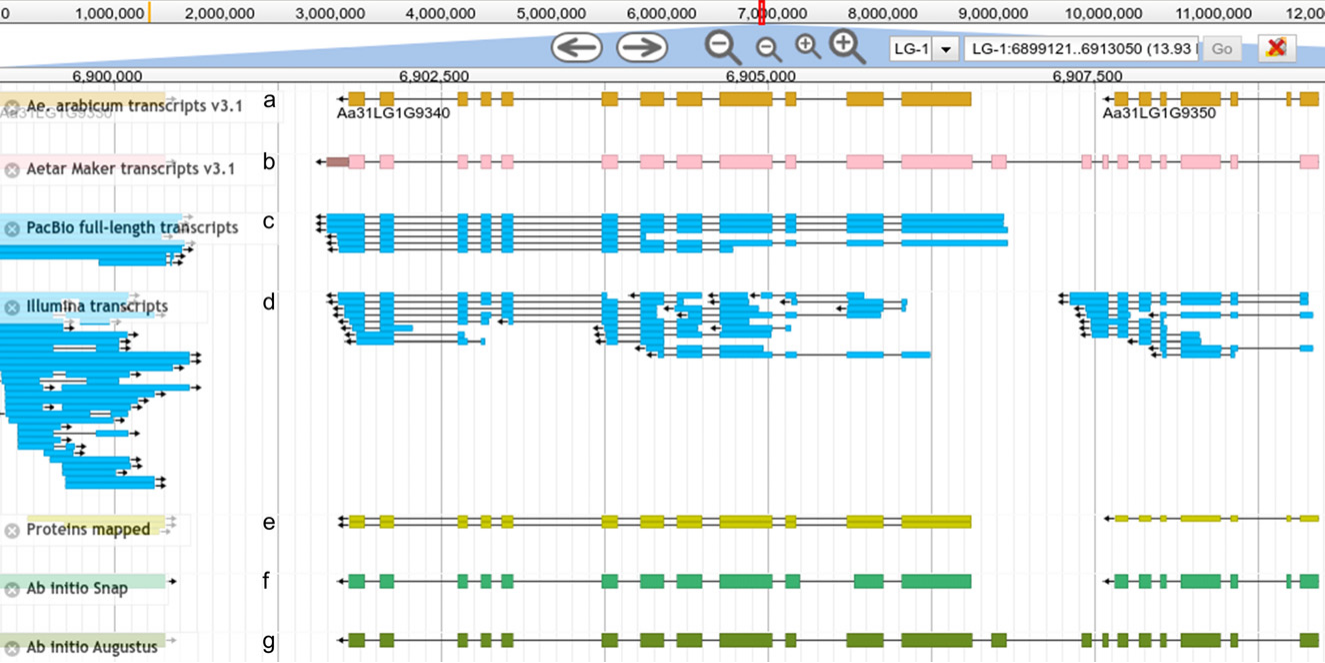
Example of a gene model incorrectly merged that was subsequently split into two genes. (a) Final gene model after split. (b) Gene model incorrectly predicted by MAKER. (c) Long‐read RNA‐seq evidence based on PacBio full‐length transcripts. (d) Short‐read RNA‐seq based on Illumina Scallop assembly. (e) Protein evidence based on Embryophyta proteins in Swiss‐Prot. (f) SNAP *ab initio* prediction. (g) Augustus *ab initio* prediction.

**Figure 4:**
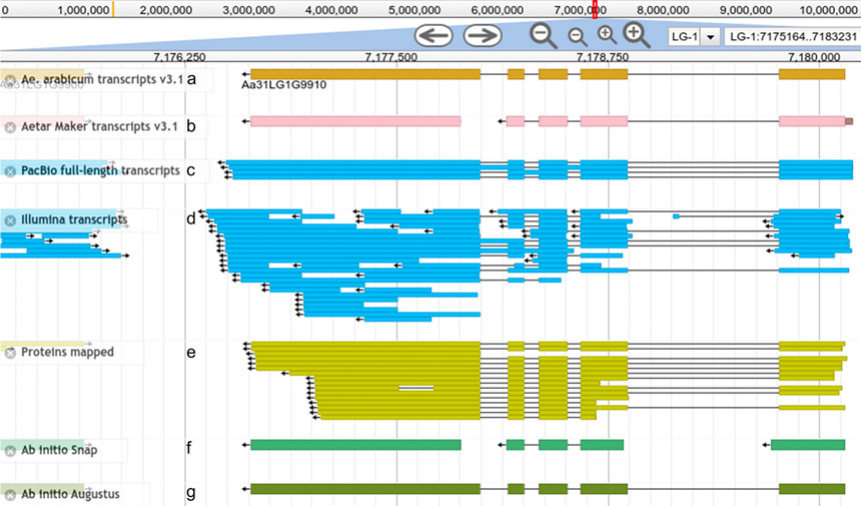
Example of two gene models incorrectly predicted that were subsequently merged into a single gene. (a) Final gene model after merging two genes. (b) Gene models incorrectly predicted by MAKER. (c) Long‐read RNA‐seq evidence based on PacBio full‐length transcripts. (d) Short‐read RNA‐seq based on Illumina scallop assembly. (e) Protein evidence based on Embryophyta proteins in Swiss‐Prot. (f) SNAP *ab initio* prediction. (g) Augustus *ab initio* prediction.

Automated annotation is not perfect, but manual curation is very time consuming and requires a large community effort to fix errors in predicted gene model structures. To try to identify abundant errors with similar sources, and thus fix them in an automated way, we implemented a genome browser with the annotation produced by MAKER and tracks displaying the data used in the annotation process (see the *Ae. arabicum* genome database below for more information). During the evaluation of the annotation, it was observed that tandem gene duplications and promiscuous protein domains often caused problems, fusing multiple genes into one or splitting genes into several parts. Manual inspection of the results followed by analysis with BedTools intersect (Quinlan and Hall, 2010) and AWK commands showed that a few hundred gene models were predicted incorrectly, and that several proteins and PacBio full‐length transcripts supported splitting some models into two different genes (Figure 3). *Vice versa*, sometimes proteins and long reads supported a single gene while MAKER decided on two separate ones (Figure 4). As an example of the first case, MAKER predicted a gene model (Figure 3b) based on Augustus *ab initio* prediction (Figure 3g). However, RNA‐seq evidence from transcripts (Figure 3c,d), proteins (Figure 3e) and SNAP *ab initio* prediction (Figure 3f) supported two gene models instead of one. In all similar cases, the gene models were split into two or more genes based on the previous gene version (Figure 3a) and protein evidence (see Experimental Procedures). In total, 426 genes were split into 909 gene models. For cases similar to the example in Figure 4, 540 gene models were merged to form 261 genes, resulting in a total of 24 849 genes.

### Final post‐processing

After the split and merge step, 83 gene models from v3.0 supported by Embryophyta Swiss‐Prot proteins but not predicted by MAKER because of insufficient support from expression data were detected. These genes were added to the v3.1 gene model annotation, obtaining a total of 24 932 genes. Adding these genes together with the split and merged ones increased BUSCO completeness by 0.7%, supporting that these steps improved the annotation. The final BUSCO completeness was 97.0% for the Embryophyta dataset odb10 and 98.8% for Viridiplantae odb10, which is close to the values for *A. thaliana* (TAIR10), 99.7% in both cases. The final gene set was named according to the previously introduced nomenclature (Nguyen *et al*., 2019). For example, in the locus name ‘Aa31LG1G10’, ‘Aa’ stands for *Ae. arabicum*, ‘31’ for gene annotation version 3.1, ‘LG’ for linkage group (or ‘sc’ for scaffold), followed by the number of the LG (linkage group 1 in the example), and ‘G’ for gene followed by the gene number (Gene 10 in the example). Gene numbers start with 10 for the first gene at the 5′ end of each LG or scaffold and increase by 10 for every gene found in the 3′ direction as in the convention for *A. thaliana*. This allows to use the free numbers between tens if needed in future annotations. Once the names were modified in the GFF file of the final v3.1 annotation, putative transposable elements (TEs) were identified and sequence files were created for cDNA (transcript sequences), gene‐coding sequences (CDSs) and proteins (Figure 1).

### Alternative splicing comparison between PacBio full‐length and MAKER isoforms

The number of isoforms based on MAKER output and PacBio full‐length transcripts was calculated (Figures S2 and S3). AS classification based on exon skipping (ES), alternative acceptors (AAs), alternative donors (ADs), mutually exclusive exons (MXs) and intron retention (IR) was done using Astalavista (Foissac and Sammeth, 2007) (Table 2, Figures S2 and S3). The number of isoforms produced by MAKER is very different from the number based on PacBio full‐length isoforms (Table 2). MAKER seems to be very conservative when creating alternative isoforms and only a few isoforms per gene are included in MAKER AS (Figure S2a). Most of the isoforms in MAKER are limited to canonical splicing sites and rules (90.1%), while a huge number of AS events is observed in the PacBio full‐length transcripts that are not canonical (Table 2, Figures S2 and S3). Moreover, 589 out of 789 (74.7%) canonical AS events in MAKER isoforms were observed in UTRs (Figure S2c). PacBio full‐length transcripts show more isoforms with changes in the CDS: only 616 out of 2240 (27.5%) canonical AS events in the PacBio full‐length transcripts were observed in the UTRs (Figure S3c). MAKER needs to support the gene models with multiple evidence and *ab initio* predictors following a set of rules that tries to predict genes based on canonical splice sites by default. As many of the PacBio full‐length transcripts could not be explained by canonical splicing rules, MAKER ignored much of the information of the PacBio full‐length transcripts during the isoform prediction. Therefore, we included the MAKER transcripts with isoforms and PacBio full‐length transcripts in the *Ae. arabicum* DB for downloading and for inspection in the genome browser.

**Table 2:** Alternative splicing (AS) identification using Astalavista

	Total AS events^a^	Total AS canonical^b^	Classified AS^c^	Classified AS canonical^2^
PacBio FL	2 851 417	21 642 (0.8%)	51 385	2240 (4.4%)
MAKER	3996	3601 (90.1%)	863	789 (91.4%)

^a^ Total AS includes all forms of AS.

^b^ Canonical refers only to events in canonical splicing sites following canonical rules of splicing.

^c^ Classified AS includes only AS due to exon skipping, alternative acceptors, alternative donors, mutually exclusive exons, and intron retention.

### *Aethionema arabicum* genome database

The annotation data produced in this study and the *Ae. arabicum* genome sequence V3.0 are available via a web‐accessible database. The *Ae. arabicum* DB (https://plantcode.online.uni‐marburg.de/aetar_db) contains tools to search annotations and genes by keywords, to download sequences and annotations from a list of genes, to look up older gene versions and *A. thaliana* orthologs and to perform BLAST searches. Its code and tools are based on previous databases such as OliveTreeDB (Jiménez‐Ruiz *et al*., 2020), the *Physcomitrella patens* Gene Model Lookup DB (Fernandez‐Pozo *et al*., 2020) and the Sol Genomics Network (Fernandez‐Pozo *et al*., 2015).

A genome browser with all evidence tracks used in this study and the expression data of the light experiments from Mérai *et al*. (2019) is available to allow manual inspection of the gene models (https://plantcode.online.uni‐marburg.de/jbrowse). Additionally, a lookup table with previous versions, *A. thaliana* genes, overlapping PacBio full‐length reads and other annotations is available in the *Ae. arabicum* DB download section.

Moreover, these data, together with previous annotation versions, genome assembly versions and data of many experiments done in *Ae. arabicum*, such as single nucleotide polymorphisms (SNPs) and RNA‐seq and BS‐seq data, are available in CoGe (Lyons and Freeling, 2008), and data from future experiments based on genome V3 will be uploaded at https://genomevolution.org/coge/SearchResults.pl?s=aethionema%20arabicum&p=genome.

### Completeness assessment

The gene annotation of *Ae. arabicum* v1.0 was lifted over to v2.5, and when the genome assembly V3 became available, the gene annotation from v2.5 was lifted over to v3.0. In the lift‐over process from v1.0 to v3.0, 987 gene models were lost (Nguyen *et al*., 2019). Additionally, the lift‐over methods carried over many interrupted open reading frames (ORFs) in both versions, 2.5 and 3.0 (Table 3), migrating genes with premature stop codons or missing the beginning or the end of the ORF. Additionally, the GFF from v2.5 and v3.0 had formatting problems, such as missing features or CDSs that were not multiples of three, which made it impossible to extract some gene sequences using standard GFF parsers such as gffread (http://ccb.jhu.edu/software/stringtie/gff.shtml#gffread). In total, 3549 genes in v2.5 and 3365 in v3.0 were affected by any of these issues (Table 3).

**Table 3:** Comparison between *Aethionema arabicum* gene versions

	v2.5	v3.0	v3.1
Raw gene number	23 594	22 607	24 932
Interrupted ORFs	3549	3365	0
Correct in GFF^a^	19 298	19 242	24 932
Putative TEs^b^	1224	1224	1772
Total correct genes^c^	18 074	18 018	23 160
Protein domains associated	–	20 926	25 703
GO terms associated	–	19 932	24 658
TAIR10 homologs^d^	–	15 804	18 826
BUSCO completeness	85.4%	85.4%	97.0%

^a^ Correct in GFF refers to the genes that were exportable from the GFF using scripts such as gffread and that did not contain obvious annotation errors.

^b^ This refers only to genes labeled as putative TEs, not to all TEs in the genome.

^c^ Total correct genes refer to correct genes in GFF without putative TEs.

^d^ BLASTp with 50% or more identity percentage and coverage length.

Additionally, analysis of protein domains with InterProScan (Jones *et al*., 2014) detected 947 genes in v3.0 containing TE domains such as ‘Reverse Transcriptase’ (PF00078, PF07727, PF13456, PF13966), ‘Integrase’ (PF00665, PF13976), ‘Retrotransposon’ (PF03732, PF08284) or ‘LTR’ (PF14223, PF14244). For that reason, BedTools intersect (Quinlan and Hall, 2010) was used to identify genes overlapping 50% or more with TE sequences predicted by TEannot (REPET package). Based on this, 1224 genes from v3.0 and 1772 genes from v3.1 were labeled as putative TEs (Table 3). In total, 5520 genes in v2.5 and 4589 genes in v3.0 were affected by obvious annotation errors or annotated as possible TEs. The annotation v3.1 created in this study using MAKER generated 24 932 genes, of which 1772 were identified as putative TEs; 721 (40.69%) in linkage groups (LGs) and 1051 (59.31%) in unassigned scaffolds, showing an enrichment of these elements in the genome fraction not included in LGs, which represent 34.5% of the genome sequence. If putative TEs are not considered in this comparison of annotation versions, there are 23 160 genes left in v3.1, 5086 genes more than in v3.0 and 5142 more than in v2.5. Moreover, the genes of v3.1 are supported by higher numbers of proteins domains, GO terms and *A. thaliana* homologs and higher BUSCO completeness, increasing the BUSCO completeness of previous versions by 11.6% (Table 3). In addition, annotations from v2.5 and v3.0 had no UTRs annotated. In v3.1, 10 841 genes feature 5′ UTR annotations, 12 522 genes have 3′ UTR annotations, and 9437 genes have annotations of both UTRs. The annotation v3.1 including long‐read transcripts allows a better detection of proximal regulator elements, like introns in the 3′ UTRs. For example, the transcript of Aa31LG1G8330, which encodes the eukaryotic release factor 1‐1 (eRF1), harbors an intron 124 bp downstream of the stop codon that mediates a regulatory role through the non‐sense mediated decay machinery in plants (Nyiko *et al*., 2017). The 3′ UTR intron was found in the v3.1 annotation while it was not annotated in v2.5 or v3.0.

The use of bioinformatics tools such as GeMoMa (Keilwagen *et al*., 2016) and Flo (Pracana *et al*., 2017) to lift over genes from annotation v1.0 to v3.0 was very useful to compare annotations and to provide a core of gene references for protein evidence in MAKER. However, using this method to migrate annotations to the next genome version has some disadvantages in comparison with *de novo* annotation pipelines. For example, in annotation v3.0 it was not possible to identify genes in regions added to genome assembly V3 (Nguyen *et al*., 2019), as it could only migrate genes in regions that already existed in previous genome sequence versions. Moreover, new expression data and proteins accumulate every year in sequence databases such as the Sequence Read Archive (SRA) (Kodama *et al*., 2012) or UniProt (UniProt, 2019). However, lift‐over annotations are frozen in time; they just move existing annotations but do not make use of accumulated new evidence in databases. When using annotation pipelines such as MAKER, it is possible to include expression data and proteins that were not available when the previous gene annotation was created.

### Predicted gene models not found in previous versions and broken genes in v3.0

Of the 18 018 genes free of obvious annotation errors and putative TEs in v3.0, 17 765 genes (98.6%) were found using BLAST versus the v3.1 protein set with a query coverage and identity percentage equal to or higher than 50%. Only six genes had no hits, and 247 had a query coverage or identity percentage below 50%. Using BedTools intersect, 17 639 genes (97.9%) overlapped on the same strand between versions 3.0 and 3.1, with 50% or more of the length of the smaller gene. Only 368 genes from v3.0 did not overlap, and 11 overlapped less than 50%. Protein sequences of v3.0 have an average length of 445 amino acids. The proteins of v3.0 that did not overlap with those of v3.1 have an average length of 334 amino acids, which is more than 100 amino acids lower than average. MAKER did not show good support of proteins or expression data for these 368 potentially incorrectly predicted genes.

**Figure 5:**
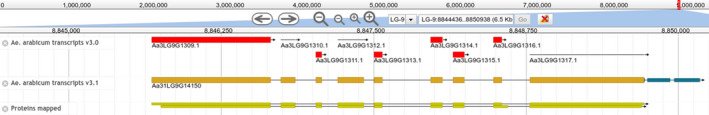
The v3.1 gene model Aa31LG9G14150 joins nine genes that had formatting or sequence errors in v3.0. The protein track shows the v3.1 gene is supported by the protein evidence.

**Figure 6:**
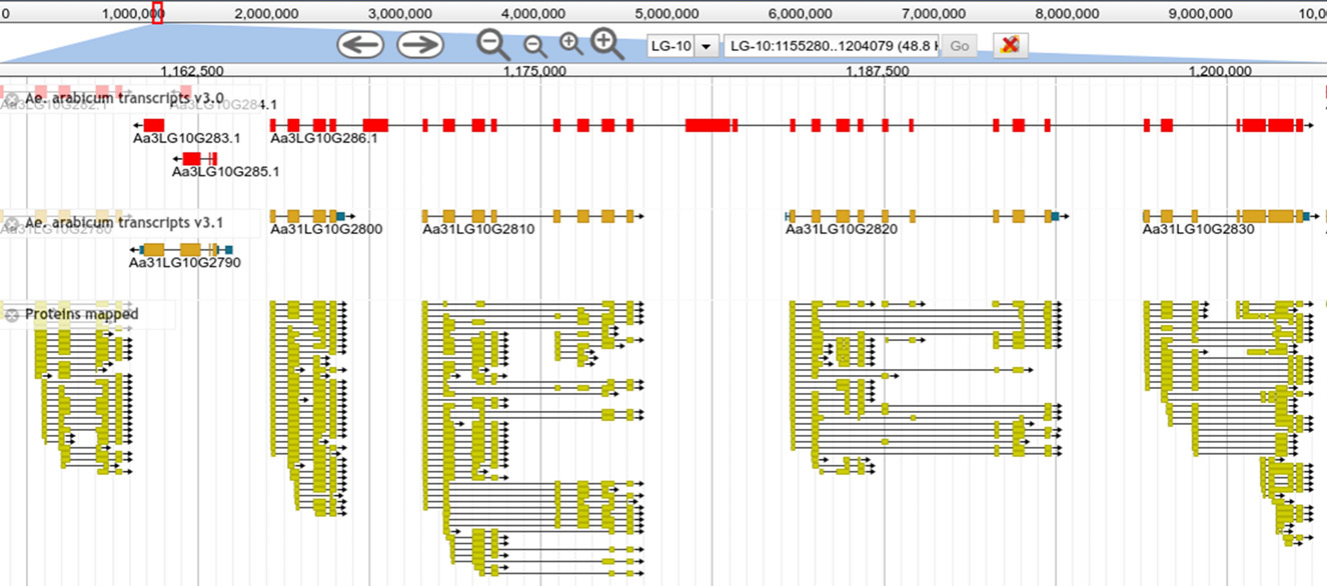
The incorrectly annotated gene model Aa30LG10G286 from v3.0 was subsequently split into four genes in v3.1. The protein track shows that the v3.1 genes are supported by protein evidence.

Of the 23 160 genes in v3.1, 5606 did not overlap with the genes of v3.0 (only 18 018 genes of v3.0 were used, putative TEs and sequences fragmented or with formatting errors were not included). Of these genes, 2878 (51.3%) are newly predicted and were not present in v3.0, and 2728 (48.7%) correspond to the fixed version of broken genes in v3.0 (81.1% of the 3365 genes broken in v3.0). Of these genes, 1945 (71.3%) were recovered as one‐to‐one relationships (see Supplemental Dataset 1). In the remaining cases, multiple genes of v3.0 were joined to generate one gene model in v3.1, while one gene of v3.0 was split in multiple genes in v3.1. For example, nine genes of v3.0 including sequence and GFF format errors were recovered in a single gene with nine exons in v3.1, Aa31LG9G14150 (Figure 5). As another example, the gene Aa3LG10G286 of v3.0 is 37 kbp long and was split into four genes in v3.1 (Figure 6). The remaining 637 broken genes were not well‐supported gene models and were not considered as valid models for v3.1 by MAKER.

### Orthology comparison with other Brassicales

*Aethionema arabicum* was compared with other Brassicales species to identify orthologous and paralogous sequences and to assess completeness and conservation of the gene models predicted in this study. The protein sequences of *Ae. arabicum*, *A. thaliana*, *Capsella rubella*, *Eutrema salsugineum* and papaya (*Carica papaya*) were analyzed with OrthoFinder (Emms and Kelly, 2019). These species represent four Brassicaceae from different genera and *C. papaya* (Brassicales), sister to the Brassicaceae, as outgroup (Figure S4).

**Figure 7:**
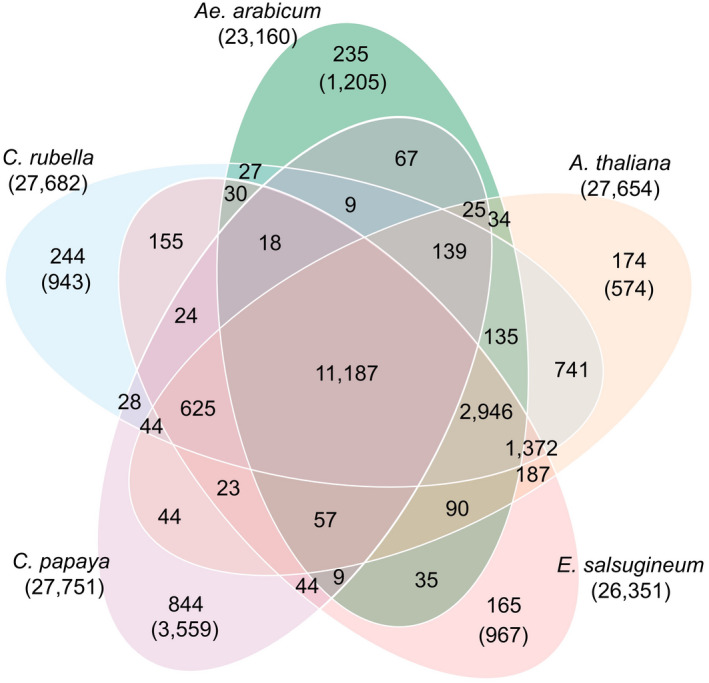
Venn diagram of orthogroups of Brassicales species. Orthologs and paralogs of *Ae. arabicum*, *A. thaliana*, *C. rubella*, *E. salsugineum* and *C. papaya* were identified using OrthoFinder. Numbers in the Venn diagram show OG counts for the intersection of every group of species. Total gene numbers are displayed in parentheses under the species name. The numbers of species‐specific genes are shown in parentheses under the number of exclusive OGs.

A Venn diagram of orthogroups (groups of genes, orthologs and paralogs, descended from single genes in the last common ancestor) identified by OrthoFinder (Figure 7) showed concordance with the topology of the species’ phylogenetic tree (Figure S4), with a decreasing number of common orthogroups (OGs) in accordance with the divergence time of the species. A total of 11 187 OGs contain genes for all of the five Brassicales, 2946 OGs include proteins of the Brassicaceae, 1372 OGs include the Brassicaceae except for *Ae. arabicum* and 741 OGs include Brassicaceae after excluding *Ae. arabicum* and *E. salsugineum*.

**Figure 8:**
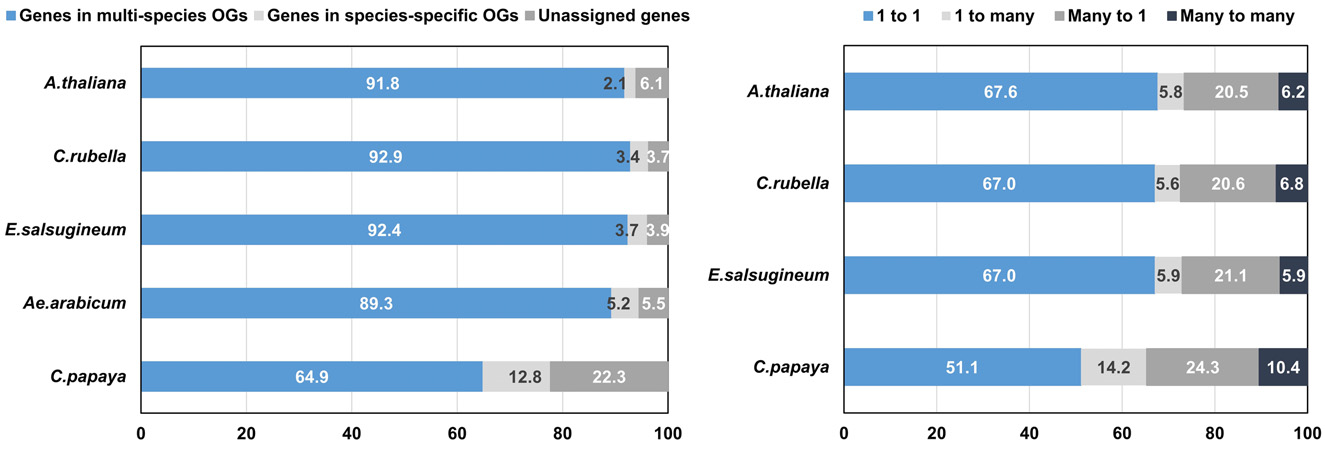
OrthoFinder genes in orthogroups and ortholog relationships for *Ae. arabicum* genes. Left: percentage of genes in OGs (blue), in species‐specific OGs (light gray) and unassigned (dark grey). Right: the relationship between orthologs of Brassicales species and *Ae. Arabicum*.

In *Ae. arabicum* 235 exclusive OGs were identified, including 1205 species‐specific genes and 1268 genes not assigned to orthogroups (Supplemental Dataset 1). Other Brassicaceae such as *C. rubella* have a similar number of exclusive OGs (244), while *A. thaliana* has a higher number of unassigned genes (1687). *Carica papaya* (Brassicales) has a higher number of exclusive genes (3559), exclusive OGs (844) and unassigned genes (6200) than any Brassicaceae (Figures 7 and 8). As expected, species that diverged less time ago have more similarity in their gene composition, with most exclusive genes found in papaya, followed by *Ae. arabicum*. Further in accordance with phylogeny, values for *Ae. arabicum* are more similar to the other Brassicaceae species than to papaya, with 89.3% of genes in OGs shared with other species in comparison with 92.4% on average in the other Brassicaceae, and only 64.9% in papaya (Figure 8, left). These results are consistent with the two rounds of unique WGDs in the Brassicaceae compared to papaya, the older At‐beta event and the At‐alpha event that occurred near the origin of the Brassicaceae, followed by the divergence of the genus *Aethionema* from the ‘crown group’ of Brassicaceae (Schranz *et al*., 2012; Walden *et al*., 2020). *Carica papaya* does not share either of the two Brassicaceae‐specific WGD events (Ming *et al*., 2008).

Additionally, the Brassicaceae species showed in 5.8% of the cases that a single gene corresponds to many in *Ae. arabicum*, while in 20.7% of the cases many genes were orthologs of only one gene in *Ae. arabicum* (Figure 8, right). The *A. thaliana* genes in many‐to‐one OGs were found to be ohnologs (homeologs) derived from the At‐α WGD in 38.87% of the cases based on the classification by (Thomas *et al*., 2007), and they were classified as tandem duplicates in 49.00% of the cases based on SynMap2 synteny analysis performed in CoGe (Lyons and Freeling, 2008). In total, 74.03% of the genes with more copies in *A. thaliana* and one copy in *Ae. arabicum* were classified as At‐α ohnologs or tandem duplicates, and 13.84% were classified as both. In the SynMap2 results, *Ae. arabicum* had 5357 tandem duplicates, a significantly (*P* < 0.01, Fisher’s exact test) lower number than in *A. thaliana* (with 10 390). The lower number of genes in *Ae. arabicum* in comparison with the other Brassicaceae could be partially due to more genome fractionation (duplicate gene loss or diploidization) in the lineage leading to *Aethionema*, compared to the crown group after the last polyploidization event (At‐α) (Walden *et al*., 2020), and a lower rate of genes generated by tandem duplication, in comparison with *A. thaliana*. Other reasons for the lower number of genes in *Ae. arabicum* could be the lack of detection of some genes in the annotation because the available expression data are not rich enough in experimental conditions and diversity of tissues or, in some cases, the concatenation of close genes. Increasing the diversity of tissues and experimental conditions in future expression samples might help to identify more genes in *Ae. arabicum*.

For the 1205 *Ae. arabicum* genes classified as species‐specific by OrthoFinder (see Supplemental Dataset 1), 157 genes were annotated with F‐box domains, which, among several functions, participate in seed germination and dormancy in interaction with plant hormones such as gibberellin (GA) or abscisic acid (ABA) (McGinnis *et al*., 2003; Peng *et al*., 2012; Song *et al*., 2012; Majee *et al*., 2018). Other species‐specific genes were annotated as cytochrome P450 (27), cysteine/histidine‐rich C1 (32), domain of unknown function (DUF) (76), glycoside hydrolase (22), NAC domains (14), peptidases (40), protein kinases (15), WRKY (15), and zinc finger proteins (35). Many of these putatively function in development and defense (Hwang *et al*., 2014; Singh *et al*., 2014; Xu *et al*., 2015; Dong *et al*., 2019) or might be related to seed germination and dormancy (Papi *et al*., 2000; Christianson *et al*., 2009; Joseph *et al*., 2014; Santamaria *et al*., 2014; Raineri *et al*., 2016; Zhou *et al*., 2020), as expected as many seed samples (234 of 294) were included for RNA‐seq data used in this annotation. Since *Ae. arabicum* is an interesting model to study seed germination and dormancy, genes in this functional context classified as species‐specific could be very useful for future studies.

### Transcription associated proteins

Transcription associated proteins (TAPs), that is, transcription factors (TFs) and transcriptional regulators (TRs), were identified using TAPscan (Wilhelmsson *et al*., 2017). A total of 2579 TAPs were identified in genome annotation v3.1, 449 more than in v3.0 (see Supplemental Dataset 2 for more information). Of those found only in v3.1, 109 do not have a clear family assignment (potentially representing unusual domain compositions). There are 18 ABI3/VP1, 18 AP2/EREBP, 32 basic helix‐loop‐helix (bHLH), 15 bZIP, 14 MADS, 16 MYB‐related, 13 MYB, 12 NAC, 10 TRAF and 24 WRKY proteins more than in v3.0. Fewer TAPs were found in v3.1 than in v3.0, i.e., 1 CAMTA, 1 CSD, 1 HD_KNOX1, 4 HSFs and 1 PcG_EZ.

The TAPs identified in *Ae. arabicum* v3.1 were compared with those found in *A. thaliana*, *Arabidopsis halleri*, *Arabidopsis lyrata*, *Capsella grandiflora*, *C. rubella*, *Boechera stricta*, *Eutrema salsugineum*, from the Brassicaceae family, and *C. papaya* as outgroup (Supplemental Dataset 2). Based on this analysis, *Ae. arabicum* has on average 308 TAPs less than other Brassicaceae and 321 TAPs more than *C. papaya* (Figure S5). *Aethionema arabicum* has the same number of TAPs as in *A. thaliana* in 29 cases, more in 24 cases and fewer in 73 cases. In the *Ae. arabicum* v3.1 annotation, 77 WRKY TFs were found, six more than in the other Brassicaceae on average (71.25). These TFs have been reported to participate in seed germination (Raineri *et al*., 2016; Zhou *et al*., 2020). Since part of the WRKY complement was detected as species‐specific by OrthoFinder, it might represent a lineage‐specific expansion of these TFs. Similar reasons as for the orthology comparison can be applied to explain the intermediate number of *Ae. arabicum* TAPs between those of *C. papaya* and the other Brassicaceae.

### Case study on completeness and orthology of TAPs: MADS TFs

To determine the quality of the *Ae. arabicum* v3.1 annotation and to understand better why the *Aethionema* TAP number is lower in comparison to the other Brassicaceae species, we had a detailed look at MADS‐box genes. These genes have important functions in many developmental processes in angiosperms (Gramzow and Theißen, 2010; Smaczniak *et al*., 2012) and hence constitute one of the best‐studied families of genes encoding TFs in plants. Two types of MADS‐box genes, termed Type I and Type II, existed probably already in the most recent common ancestor (MRCA) of extant eukaryotes (Gramzow *et al*., 2010). In plants, Type II genes have a special domain structure and are hence termed MIKC‐type genes, comprising ‘classical’ MIKC^C^‐type as well as MIKC*‐type genes (Gramzow and Theißen, 2010). Here, we focus on MIKC^C^‐type genes, because they have a quite well‐defined phylogeny, and even gene loss has been studied comprehensively (Cheng *et al*., 2013; Gramzow and Theißen, 2015; Hoffmeier *et al*., 2018). The number of MIKC^C^‐type genes has increased during the evolution of land plants, mostly due to preferential retention and diversification of duplicates after WGDs, and much less by small‐scale duplications (Theißen *et al*., 2018). Specific MIKC^C^‐type genes thus often can be traced back to ancient polyploidization events such as the five that occurred in the evolutionary history of Arabidopsis (At‐α to At‐ζ) (Cheng *et al*., 2013).

All MIKC^C^‐type genes of flowering plants trace back to 11 seed plant‐specific superclades that were present in the MRCA of extant seed plants. These genes evolved into 17 clades that had already been established in the MRCA of extant flowering plants (Gramzow *et al*., 2014). These clades comprise SQUA‐ (AP1‐), AGL2‐ (SEP1‐), AGL9‐ (SEP3‐), AGL6‐, DEF‐ (AP3‐), GLO‐ (PI‐), AG‐ and STK‐like genes that all have conserved functions in the specification of floral meristem and organ identity. The genes of other clades, such as GGM13‐ (B_sister_‐), AGL12‐, StMADS11‐ (SVP‐), FLC‐, TM3‐ (SOC1‐), OsMADS32‐, TM8‐, AGL15‐ and AGL17‐like genes, have quite variable or more subtle functions in diverse aspects of plant development, ranging from root to fruit development, or have even unknown functions (for a review, see Gramzow and Theißen, 2010). Most clades have never been completely lost in any of the flowering plant genomes investigated so far, with the remarkable exceptions of OsMADS32‐ and TM8‐like genes, which both do not exist in Brassicaceae (Gramzow and Theißen, 2015).

We identified the complete sequence of 65 MADS‐box genes in the *Ae. arabicum* genome, with 25 Type I, 4 MIKC*‐type and 35 MIKC^C^‐type genes and one that could not be unambiguously assigned to any of these clades. Eight of these genes are not represented in the v2.5 and v3.0 gene annotation, and all but three were part of the v3.1 annotation. These three genes are one MIKC*‐type gene, a TM3‐like gene and an AGL17‐like gene. The genomic position of these three MADS‐box genes was found to overlap with hard‐masked repeat regions, which is probably the reason why they have not been included in the annotation. Overall, the identification of MADS‐box genes in *Ae. arabicum* v3.1 is nearly complete.

Detailed comparison of the gene models in *Ae. arabicum* v3.1 to their orthologs in *A. thaliana* revealed that 30 of the models fit very well to the *A. thaliana* orthologs. For 32 genes, an improvement of the fit to the *A. thaliana* orthologs was reached by a novel gene annotation using the genomic sequence of *Ae. arabicum* and the protein sequence of an ortholog from another Brassicaceae species as input for the fgenesh+ program (Salamov and Solovyev, 2000). Most of the genes for which the annotation was improved are MIKC*‐type and MIKC^C^‐type genes, which usually have 6–10 exons and are difficult to annotate correctly.

In the following, we focus exclusively on MIKC^C^‐type genes for a detailed analysis of the identified MADS‐box genes. For the clades of AGL2‐, AGL9‐, STK‐, AG‐, AGL12‐, DEF‐, GLO‐, GGM13‐, StMADS11‐, TM3‐ and AGL17‐like genes, we found orthologs in *Ae. arabicum* for all *A. thaliana* genes as expected (Figure S6, Dataset S2).

In *A. thaliana*, there are four SQUA‐like genes, which all originated at At‐α or earlier, so that one may expect orthologs in *Aethionema*. We identified five SQUA‐like genes (Figure S6, Dataset S2) as one‐to‐one orthologs to *AGL79*, *CAL* and *AP1* and two co‐orthologs to *FRUITFUL* (*FUL*). However, the genomic region where the two FUL‐like genes in *Ae. arabicum* are encoded are identical for several thousand bases, which might represent an assembly artifact. Remarkably, the *FUL* and *AGL79* orthologs were not identified in the v2.5 annotation of *Ae. arabicum*, even though expression of the *FUL* gene had previously been shown in the literature (Lenser *et al*., 2016). Seven MADS‐box genes not found in previous versions were identified in v3.1; among them are the orthologs of *FUL* and *AGL79*. These genes have important functions in *A. thaliana*, so their annotation in *Ae. arabicum* represents considerable progress. During Brassicaceae development, *FUL* is first involved in a redundant way together with *AP1* and *CAL* in the establishment of floral meristem identity, but the gene has a second, non‐redundant function in leaf and fruit development. For example, in the *ful* mutant, a lack of proper differentiation of cells in the fruit walls compromises fruit elongation. Therefore, some fruits rupture prematurely as the seeds develop (Gu *et al*., 1998). The function of *AGL79* is less well understood. The gene appears to be broadly involved in developmental control of lateral roots, shoot branching and leaf shape (Gao *et al*., 2017).

There are two AGL6‐like genes in *A. thaliana*. We also identified two AGL6‐like genes in *Ae. arabicum*, which both seem to be co‐orthologs of *AGL13* according to our preliminary phylogeny. Consequently, we did not identify the *AGL6* ortholog (Figure S6, Dataset S2).

There are six FLC‐like genes in the *A. thaliana* genome. In *Ae. arabicum*, one FLC‐like and two MAF‐like genes had already been identified previously (Theißen *et al*., 2018), of which we have now identified one FLC‐ and one MAF‐like gene (Figure S6, Dataset S2). However, there is a sequencing gap in the middle of the genomic locus where the MAF‐like gene of *Ae. arabicum* is encoded. Hence, it is possible that also at this locus two MAF‐like genes are encoded of which parts are ‘hidden’ by the sequencing gap. With one FLC‐ and one MAF‐like gene, *Ae. arabicum* has a lower number of FLC‐like genes than *A. thaliana*, where six FLC‐like genes are identified. However, five of the *A. thaliana* genes trace back to tandem duplications in the MAF gene subfamily after the lineage that led to *Ae. arabicum* had already branched off (Theißen *et al*., 2018). This is an example of a gene family with more genes in other Brassicaceae than in *Ae. arabicum* that expanded by tandem duplications.

For the two AGL15‐like genes of *A. thaliana*, we found only one *Ae. arabicum* ortholog for *AGL18* but none for *AGL15* (Figure S6, Dataset S2).

### Alternative splicing of seed dormancy genes

Seeds can sense light, water and temperature to control germination by the antagonistic regulation of ABA and GA (Rodriguez‐Gacio Mdel *et al*., 2009). The induction and maintenance of seed dormancy is achieved by increased ABA levels and sensitivity and decreased GA levels and sensitivity (Finch‐Savage and Leubner‐Metzger, 2006; Toh *et al*., 2008; Oh *et al*., 2009; Rodriguez‐Gacio Mdel *et al*., 2009). Mérai *et al*. (2019) showed that seeds of the TUR accession germinated well in light, while CYP seeds were strongly inhibited, making *Ae. arabicum* an excellent model to study seed germination and its response to light.

Recent work by Punzo *et al*. (2020) demonstrates that RNA splicing is a fundamental mechanism to integrate ABA sensitivity and light signaling. These authors report that the DRT111 splicing factor controls expression and splicing of genes involved in ABA responses, light signaling and mRNA splicing, including targets of ABSCISIC ACID INSENSITIVE 3 (ABI3), phytochrome A (phyA), PHYTOCHROME INTERACTING FACTOR 1 (PIF1) and PIF6 (Punzo *et al*., 2020; Thiruppathi, 2020). Consistently with the deregulation of ABI3 target genes, defective splicing of ABI3 was observed in *drt111* mutant seeds. The improved annotation v3.1, with 136 307 isoforms of the ecotypes TUR and CYP previously unknown in *Ae. arabicum*, is a valuable resource to study the role of AS in *Ae. arabicum*. As an example of ecotype‐specific AS, the genes *PIF6*, *ABI3*, *ABI4*, *DOG1* and *9‐cis‐epoxycarotenoid dioxygenase 6* (*NCED6*), which play key roles in ABA‐mediated seed dormancy, were studied.

Members of the PIF bHLH TF family are involved in the plant light response through interaction with phytochromes, temperature signaling and integration of signaling through several hormonal signaling pathways including ABA (Paik *et al*., 2017; Tripathi *et al*., 2019). PIF6 contains an APB domain, required for phyB interaction, and a DNA‐binding bHLH domain and is also implicated in control of primary seed dormancy in *A. thaliana* (Khanna *et al*., 2004; Penfield *et al*., 2010; Golonka *et al*., 2019). Both CYP and TUR accessions have at least one transcript encoding a full PIF6 protein (cyp_c24358_1_1433 and tur_c5191_1_1154) (Table S2, Figure S7). The transcripts encode an ORF with both an APB domain and a bHLH domain. Two of the transcripts are short and lack APB domains required for phyB interaction but encode proteins with a bHLH domain (tur_c13947_1_1037 and tur_c28278_1_995). Interestingly, one of the isoforms (cyp_c7226_2_1319) appears to encode a protein with an APB domain but an incomplete bHLH domain, due to skipping the fourth exon, similar to the *PIF6*‐*β* transcript in *A. thaliana*, whose overexpression is associated with reduced dormancy (Penfield *et al*., 2010). The role of ecotype‐specific splicing of *PIF6* in the ecotype‐specific seed light response is a future research subject.

*ABI3* in *A. thaliana* and *VIVIPAROUS1* (*VP1*) in cereals encode a TF that is a crucial component of the ABA signaling pathway and one of the master ABA‐related regulators of seed maturation and dormancy (Bentsink and Koornneef, 2008; Holdsworth *et al*., 2008; Suzuki and McCarty, 2008; Graeber *et al*., 2010). ABI3 has four main domains: the acidic A1 domain is responsible for co‐activation/repression activity (McCarty *et al*., 1991); the basic domain B1 is responsible for interaction with ABI5 (Nakamura *et al*., 2001); the basic domain B2 determines nuclear localization (Marella and Quatrano, 2007); and the basic B3 domain binds a highly conserved RY DNA motif (Suzuki *et al*., 1997; Monke *et al*., 2004). Three of the identified *ABI3* transcripts in *Ae. arabicum* contain sequences with all A1, B1, B2 and B3 domains (Table S3, Figure S8). However, only the transcript from the CYP ecotype (cyp_c7392_1_2236) has an ORF encoding all four domains. An ORF in the TUR ecotype (in tur_c14427_1_2553) encodes a protein with B2 and B3 domains. Another (in tur_c25735_1_1118) encodes a protein with only a B3 domain. Alternative splice forms occur also in other species. A truncated *ABI3* isoform (*ABI3‐β*) in *A. thaliana* encodes only the A1 and B1 domains and accumulates predominantly near completion of seed maturation. It is postulated to downregulate the seed maturation program (Sugliani *et al*., 2010). AS of *ABI3* is observed in pea (*Pisum sativum*) (Gagete *et al*., 2009), flax (*Linum usitatissimum*) (Wang *et al*., 2018) and tomato (*Solanum lycopersicum*), resulting in transcripts with tissue‐specific functionality (Gao *et al*., 2013). AS of *VP1* in wheat (*Triticum aestivum*) and barley (*Hordeum vulgare*) results in transcripts not encoding full‐length VP1 proteins and, as a result, a reduction in primary dormancy and sensitivity to pre‐harvest sprouting (McKibbin *et al*., 2002; Wilkinson *et al*., 2005; Graeber *et al*., 2010). Thus, it is interesting that the only transcript with a full‐length uninterrupted *ABI3* ORF containing all four domains was found in the more dormant CYP ecotype. It is also interesting to speculate that the isoforms in the TUR ecotype may serve different functions.

ABI4 represses ABA accumulation in seed germination mediated by phyA under light conditions (Barros‐Galvao *et al*., 2020). In *Ae. arabicum*, we have observed a full‐length antisense transcript of ABI4 in CYP, also supported by Illumina transcripts (Figure S9). Both sense and antisense transcripts were confirmed by strand‐dependent cDNA synthesis and PCR in TUR and CYP, whereas the spliced variants were only detected in CYP (Figure S10). In transcriptome analysis, ABI4 was significantly more highly expressed in TUR than in CYP under light conditions (Mérai *et al*., 2019), and we observed more antisense transcript in CYP than in TUR in light conditions (Figure S9).

*DOG1* is a key gene to control seed dormancy by requiring PP2C phosphatases of the ABA signaling pathway (Nee *et al*., 2017; Nishimura *et al*., 2018; Carrillo‐Barral *et al*., 2020). In *A. thaliana*, DOG1 expression is regulated by AS and polyadenylation (Nonogaki, 2019). The gene has three exons and produces five transcript variants that are translated into three distinct proteins (Nonogaki, 2017). A shorter version of DOG1 codified only by the two first exons is more effective to enhance dormancy than the longer versions with three exons, and the genomic region includes an antisense promoter that regulates the expression of an antisense transcript asDOG1, which inhibits DOG1 expression and reduces seed dormancy (Fedak *et al*., 2016; Nonogaki, 2019). In *Ae. arabicum*, we identified only one isoform with two exons for *DOG1* (Figure S11), and this has also been observed in other Brassicaceae species (Graeber *et al*., 2010, 2013). Interestingly, there is also an antisense transcript at the 3′ end of DOG1 (Figure S11). In *Ae. arabicum*, we observed this region is very rich in thymines and adenines, and full of stop codons in all frames, as in *A. thaliana*, which is conserved in other Brassicaceae at the nucleotide level but not at the protein level (Fedak *et al*., 2016). We also observed an antisense transcript of *ABI3*, showing homology (based on BLAST) to a similar antisense transcript in *A. thaliana*, although a function for this transcript is not known.

On the other hand, NCEDs catalyze an important step in ABA biosynthesis and are involved in dormancy maintenance and responses to stress such as drought (Iuchi *et al*., 2001; Lefebvre *et al*., 2006; Martinez‐Andujar *et al*., 2011). NCEDs are typically intronless (Tan *et al*., 2003; Priya and Siva, 2015); however, in *Ae. arabicum*, we observed full‐length transcripts with introns in the *NCED6* TUR accession (Figure S12). *NCED6* was differentially expressed between CYP and TUR ecotypes under light conditions (Mérai *et al*., 2019), showing lower expression in TUR, which is the less dormant ecotype (Figure S12).

Full‐length transcripts to study gene regulation mediated by AS are so far available only for a few plant species. Here, we have shown that sets of PacBio full‐length transcripts, together with well‐chosen combinations of bioinformatics tools, create a useful resource in that regard. With selected examples for genes with well‐defined biological functions, our data reveal the usefulness of complementing the growing genome sequence collection with improved full‐length transcript annotation. The datasets generated allow mining for AS in other contexts of interest and enable addressing evolutionary questions. It allows building numerous new hypotheses for experimental work, adding to our understanding of diversity between species and ecotypes and tissue and cell specificity.

## CONCLUSIONS

*Aethionema arabicum* is an interesting model plant to study seed germination because of its fruit and seed heteromorphism and its plasticity to germinate in response to different environmental conditions, showing differences between the ecotypes TUR and CYP. The annotation presented here provides a plethora of alternatively spliced full‐length isoforms of both ecotypes. Additionally, the *Ae. arabicum* gene annotation is a valuable resource for comparative genomics analyses, especially for Brassicaceae evolution studies, because *Ae. arabicum* is a sister lineage to the Brassicaceae crown group. All the datasets generated and bioinformatics tools are available at the *Aethionema arabicum* DB, allowing easy access to the data and facilitating the exploration of the annotation and full‐length isoforms in a genome browser.

## EXPERIMENTAL PROCEDURES

### Repetitive elements

To build libraries of consensus sequences that are representative of repeated sequences, the TEdenovo pipeline (Flutre *et al*., 2011) from the REPET package (v2.4) was launched on full assemblies when their size was below 300 Mb or on a subset of assemblies composed of the longest contigs to a cumulative size of 300 Mb. The minimum number of high scoring pairs for grouping was set to *n* = 3, *n* = 5 and *n* = 10 for assembly size below 150 Mb, in the 150–300 Mb range and above 300 Mb, respectively. The PASTEC utility (Hoede *et al*., 2014) was used to classify the consensus sequences followed by semi‐manual curation. For each genome, the TEannot pipeline (Quesneville *et al*., 2005) from the REPET package was launched a first time to select consensus sequences with at least one full‐length copy in the respective input genomic sequence. TEannot was launched a second time with filtered library as input to annotate respective complete assemblies. The genome assemblies used were retrieved from the following sources: *Aethionema arabicum* V3.0 (CoGe), *Aethionema arabicum* V1.0, *Sisymbrium irio* and *Leavenworthia alabamica* (http://mustang.biol.mcgill.ca:8885/), *Camelina sativa* (https://www.ncbi.nlm.nih.gov/assembly/GCA_000496875.1), *Boechera stricta* v1.2 (Phytozome v12), *Eutrema salsugineum* v1.0, *Capsella rubella* v1.0 and *Arabidopsis lyrata* v1.0 (Phytozome v11), *Brassica oleracea* (Ensembl 39), *Brassica rapa* (Ensembl 33), *Thellungiella parvula* (http://thellungiella.org/data/), *Arabidopsis thaliana* (TAIR10) and *Arabis alpina* (https://www.ncbi.nlm.nih.gov/nuccore/JNGA00000000.1).

The repeat set produced for the *Ae. arabicum* genome V3.0 by REPET was provided to MAKER to mask the genome sequence, which in the case of interspersed repeats will avoid that transcripts and proteins align to these repetitive regions and call exons. Interspersed repeats (complex) are hard‐masked to avoid portions of TEs to be incorrectly included in annotations of neighboring protein‐coding genes, and low‐complexity repeats (simple) are soft‐masked (Campbell *et al*., 2014) (http://weatherby.genetics.utah.edu/MAKER/wiki/index.php/MAKER_Tutorial_for_WGS_Assembly_and_Annotation_Winter_School_2018#Repeat_Masking).

### Long‐read sample preparation and sequencing

*Aethionema arabicum* total RNA from germinating seeds and leaves of young plants of ecotypes TUR and CYP was extracted using the CTAB protocol (Graeber *et al*., 2011). The seeds were received from Professor Leubner (SeedAdapt consortium). Plants were grown in the Marburg lab, young seedlings as well as leaves were harvested and RNA was isolated and pooled for library generation. RNA quality was analyzed using the Plant RNA Nano assay of a 2100 Bioanalyzer (Agilent Technologies, Santa Clara, CA, USA). PacBio library preparation and sequencing were performed by the Max Planck Genome‐center Cologne (MP‐GC) sequencing facility. Library preparation and size selection were done as described by (Cartolano *et al*., 2016). The large‐scale amplified cDNAs were pooled and column purified (Qiagen PCR Purification Kit; Qiagen, The Netherlands) and assessed on an Agilent Bioanalyzer DNA 12000 chip, and then three separate size ranges were fractionated on SageELF (SAGE Science, Beverly, MA, USA): 0.5–1, 1–2 and over 2 kb (Cartolano *et al*., 2016). Sequencing was performed using a Pacific Biosciences RSII sequencer for 360 min with P4 polymerase and C4 chemistry.

### Long‐read sequence analysis

PacBio ROI were processed using PacBio’s Iso‐seq ToFU tool (Gordon *et al*., 2015) and PacBio SMRT Analysis v5.1 with default settings.

Reads were classified into full‐length and non‐full‐length reads using ToFU pbtranscript classify with default settings. If primers or poly‐A tails were identified, they were removed. Full‐length reads were clustered and error corrected using ToFU pbtranscript cluster with default settings. Full‐length reads were further error corrected using LoRDEC v0.8 (Salmela and Rivals, 2014) together with Illumina short reads using lordec‐build‐SR‐graph and lordec correct with ‐k 19 ‐s 3 and default settings. Full‐length consensus sequences were mapped to the *Ae. arabicum* genome V3 and *A. thaliana* organelles and rDNA, using GMAP v2018‐03‐11 (Wu and Watanabe, 2005) to keep sequences from nuclear genes and discard rRNA and organellar RNA sequences. *Arabidopsis thaliana* references were used because there were no publicly available *Ae. arabicum* organelle and rDNA sequences.

### Short‐read sample preparation and sequencing

A total of 294 RNA‐seq samples sequenced with Illumina technology were used to create an assembled transcriptome fed to MAKER as input for expression evidence. Four samples were SE reads 100 bp long from indehiscent and dehiscent seeds (Wilhelmsson *et al*., 2019). The other 290 samples were 50 bp long SE reads, including 12 samples from a light inhibition experiment (Mérai *et al*., 2019) and 278 RNA‐seq samples not published before (see the Accession numbers section below). The 294 samples included seed (234), shoot (18), root (18), flower (8), flower bud (8) and fruit (8), under several experimental conditions.

Sample preparation and sequencing of the 278 RNA‐seq Illumina samples not published before were done as in (Mérai *et al*., 2019) with an Illumina HiSeq 2500 sequencer at the NGS Unit of the Vienna BioCenter Core Facilities. Seed, shoot, root, flower, flower bud and fruit tissues were used to produce SE 50 bp long libraries.

### Short‐read analysis

Removal of adapters, poly‐A tails, low‐quality reads, rRNA and organelle DNA was done as in (Wilhelmsson *et al*., 2019). Reads with a minimum length of 30 nt were kept after pre‐processing.

Multiple transcriptome assemblers were tested in genome‐guided mode and default options, with few exceptions, to provide a transcriptome assembly with Illumina reads as input to MAKER. Trinity v2.6.6 (Grabherr *et al*., 2011) was used with 10 000 bp max intron length. Scallop v0.10.2 (Shao and Kingsford, 2017) was used with unstranded library type. StringTie v1.3.3 assembly was used with the options ‐f 0.4, ‐a 15, ‐j 2 and ‐c 4. Merge step was performed with the option ‐f 0.3. It was performed separately four times, for the RNA‐seq libraries from seed, root, shoot and flower, respectively. The four transcriptome assemblies were unified using Gffcompare. PASA v2.3.3 (Haas *et al*., 2003) Launch_PASA_pipeline.pl was used with default options as recommended in the manual and Gmap was used as mapper to create a comprehensive transcriptome combining the PacBio full‐length and StringTie Illumina transcripts.

### MAKER genome annotation

MAKER v2.31.9 was used for structural annotation of the *Ae. Arabicum* V3 genome. As *ab initio* predictors, SNAP (Korf, 2004) and Augustus (Stanke *et al*., 2006) were used. The *A. thaliana* training set was employed since it produced better or equal annotation edit distance (AED) curves than any set of genes tested (Figure S13). REPET (Quesneville *et al*., 2005) was used to provide repeats to MAKER. Scallop transcriptome assembly and full‐length PacBio transcripts were provided to MAKER as gene expression evidence. Manually curated and annotated Embryophyta protein sequences were downloaded from Swiss‐Prot on March 6, 2018, and were used as protein evidence in MAKER. JBrowse v1.13.0 was used to manually evaluate MAKER results. BedTools was used to identify gene models overlapping with multiple proteins and genes of previous versions and *vice versa*. To merge or split these genes, AWK was used to format BedTools results and custom Python scripts were used to assign lifted‐over genes of v3.0 to replace wrong gene models of v3.1.

### Functional annotations and completeness assessment

InterProScan v5.28‐67.0 (Jones *et al*., 2014) with default values and the Pfam database v31.0 (El‐Gebali *et al*., 2019) was used to identify protein domains and GO terms. BedTools intersect (Quinlan and Hall, 2010) was used to identify genes covered by 50% or more by TE sequences predicted by TEannot (REPET package). These genes were labeled as putative TEs.

To compare gene models from v3.0 and v2.5 with homologous sequences from *A. thaliana*, BLASTp v2.2.30+ (Camacho *et al*., 2009) was used against the TAIR10 protein representative gene model dataset and AWK was used to filter hits with an identity percentage and coverage of the Arabidopsis genes of 50% or more. BLAST v2.2.30+ and BedTools v2.26.0 (Quinlan and Hall, 2010) were used for comparisons between gene model versions.

BUSCO v4.1.4 (Seppey *et al*., 2019) with the dataset Embryophyta Odb10 was used to calculate BUSCO completeness for protein sequences of *Ae. arabicum* annotations v2.5, v3.0 and v3.1 after removing sequences with evident errors such as formatting problems in the GFF, incomplete proteins, sequences with incorrect stop codons or CDSs that were not multiples of three. The final BUSCO completeness score for *Ae. arabicum* v3.1 and TAIR10 protein sets was calculated using Embryophyta and Viridiplantae Odb10. BUSCO v3.0.1 with the dataset Embryophyta Odb9 was used for evaluation of intermediate results and MAKER input transcriptome tests.

### Orthology comparison with other Brassicales

OrthoFinder (Emms and Kelly, 2019) was used with default options to identify orthologous proteins between *Ae. arabicum* v3.1, *A. thaliana* TAIR10, *C. rubella* v1.0, *E. salsugineum* v1.0 and *C. papaya* ASGPBv0.4. Protein sequences were downloaded from Phytozome v12 (Goodstein *et al*., 2012). *Arabidopsis thaliana* and *Ae. arabicum* tandem duplicates were downloaded from the SynMap2 results after comparing *A. thaliana* TAIR10 and *Ae. arabicum* v3.1 with themselves respectively. SynMap2 was used to calculate Ks with a CodeML max value of 3 and default options.

### Transcription associated protein analysis

Brassicales species were downloaded from Phytozome v12 (Goodstein *et al*., 2012). TAPs from *Ae. arabicum* and Brassicales species were analyzed using TAPscan as in (Wilhelmsson *et al*., 2017).

### Analysis of MADS‐box genes

To identify MADS‐box genes, the *Ae. arabicum* genome was translated in all six reading frames into conceptual protein sequences. The translated genome was searched using a Hidden Markov Model (Eddy, 2011) for the MADS domain as described in (Gramzow and Theissen, 2013). When a MADS domain was identified, gene prediction was conducted on the corresponding genomic locus including 5000 bp up‐ and downstream using Augustus (Stanke *et al*., 2006). The CDSs of the MADS‐box genes found this way were used as query sequences in a BLASTn search (Altschul *et al*., 1990) against the set of CDSs in the annotation *Ae. arabicum* v3.1 to investigate which MADS‐box genes were already present in the annotation. Furthermore, the conceptual protein sequences of the MADS‐box genes identified on the genomic sequence were also used as query sequences for BLASTp searches on NCBI (Boratyn *et al*., 2013) to investigate the fit of the predicted proteins in *Ae. arabicum* to orthologs from other species. If there were differences between the predicted proteins in *Ae. arabicum* and the orthologs from other species, a MADS‐box gene prediction was done using the fgenesh+ program (Solovyev *et al*., 2006) with an ortholog from another species as guide and the gene‐finding parameters of ‘Dicot plants, Arabidopsis (generic)’. To assign the identified MADS‐box genes to the known clades, phylogenies were reconstructed. CDSs of MADS‐box genes from *Ae. arabicum*, *A. thaliana*, *B. rapa*, *C. papaya*, *Populus trichocarpa*, *Vitis vinifera* and *Oryza sativa* (Arora *et al*., 2007) were combined. CDSs were translated into protein sequences using transeq (Rice *et al*., 2000) and aligned using Probalign (Roshan and Livesay, 2006). The alignment was trimmed using trimAl (Capella‐Gutierrez *et al*., 2009) with the options ‐gt 0.9 ‐st 0.0001 and the trimmed alignment was trimmed once again using the options ‐seqoverlap 70 and ‐resoverlap 0.7. Based on this trimmed alignment, an unrooted RAxML phylogeny (Stamatakis, 2014) was reconstructed on the CIPRES portal (Miller *et al*., 2011). Type I and Type II MADS‐box genes were separated on the resulting phylogeny and another unrooted phylogeny was reconstructed for Type II MADS‐box genes. To do so, CDSs of Type II MADS‐box genes were translated into protein sequences using transeq and aligned using probalign, and the alignment was trimmed using trimAl as before. The phylogeny was reconstructed using RAxML as described above. The phylogeny was formally rooted on the split between MIKC* and MIKC^C^ group genes as supported by previous studies (Henschel *et al*., 2002). Based on this phylogeny, *Ae. arabicum* MADS‐box genes were assigned to the different clades.

### Strand‐dependent cDNA synthesis and PCR of TUR and CYP ABI4

For RNA samples, 30 mg of seeds of *Ae. arabicum* TUR and CYP was illuminated for 23 h with 100 µmol m^−2^ s^−1^ continuous white light at 14°C, as described in Mérai *et al*. (2019). RNA was extracted as described by (Onate‐Sanchez and Vicente‐Carbajosa, 2008). Then, 200 ng of DNase‐treated RNA was used for cDNA synthesis using the RevertAid kit (Thermo Fisher Scientific, Wilmington, DE, USA). cDNA was synthetized using either forward (5′‐ATGGACCCTTTCATCTCCCAAG‐3′) or reverse (5′‐ACCGGTTGAGATCCATCTCCC‐3′) primers specific to *AearABI4* (Aa31LG5G19360). Sense and antisense transcripts were detected using Phusion polymerase (Thermo Fisher Scientific) with the following primers: forward (5′‐CGGTCCAGACAACGCTAAAT‐3′) and reverse (5′‐AAGACGTCGGAACATCAGGT‐3′).

### Alternative splicing analysis

AS events were calculated with Astalavista (Foissac and Sammeth, 2007) using default options. Canonical splicing events were calculated including the options CSS to consider introns only with canonical splice sites and IOK to consider only basic splicing rules. ES, AAs, ADs, MXs and IR were parsed from Astalavista results.

### Accession numbers

Data from a total of 294 RNA‐seq experiments with Illumina short reads were used. Four experiments of 100‐bp SE reads for indehiscent and dehiscent seeds (PRJNA413671) and 290 experiments of 50‐bp SE reads from multiple tissues and several experimental conditions are available at NCBI SRA (PRJNA517709, PRJNA611900, PRJNA612493, PRJNA639399, PRJNA639669, PRJNA639786). PacBio Iso‐seq reads for the *Ae. arabicum* ecotypes CYP and TUR have been made available under the BioProject PRJNA639924.

## AUTHOR CONTRIBUTIONS

NFP and SAR conceived the work, supervised it and wrote the manuscript with support of all the authors. NFP and TM performed the bioinformatics analysis. FM did the repeat masking of genome V3. JOC, NFP, ZM and GLM analyzed seed dormancy genes. NFP, MES and SAR analyzed TAPs and compared *Ae. arabicum* with orthologs and TAPs in Brassicales. LG and GT analyzed MADS‐box genes. ZM and OMS contributed RNA‐seq data, managed Illumina sequencing and analyzed eRF1 locus and seed dormancy genes.

## CONFLICT OF INTEREST

The authors declare no conflicts of interest.

## Supporting information


**Figure S1**. Repetitive content across Brassicaceae.
**Figure S2**. Alternative splicing in MAKER isoforms.
**Figure S3**. Alternative splicing in PacBio full‐length isoforms.
**Figure S4**. Phylogenetic relationships of the Brassicales species included in OrthoFinder and TAP analysis.
**Figure S5**. Count of transcription associated proteins (TAPs) of *Ae. arabicum* in comparison with other Brassicales.
**Figure S6**. Phylogeny of Type II MADS‐box genes from *Ae. arabicum* and other representative flowering plant species.
**Figure S7**. *PIF6* alternative splicing isoforms in *Ae. arabicum*.
**Figure S8**. *ABI3* alternative splicing isoforms in *Ae. arabicum*.
**Figure S9**. *ABI4* isoforms and expression in the *Ae. arabicum* DB genome browser.
**Figure S10**. Strand‐dependent cDNA synthesis and PCR analysis for sense and antisense strands of *ABI4* in TUR and CYP.
**Figure S11**. *DOG1* alternative splicing isoforms in *Ae. arabicum* (A) and *A. thaliana* (B) shown in the *Ae. arabicum* DB and TAIR genome browsers, respectively.
**Figure S12**. *NCED6* isoforms in the *Ae. arabicum* DB genome browser.
**Figure S13**. Annotation edit distance curves for several training sets for SNAP and Augustus.
**Table S1**. PacBio sequencing statistics.
**Table S2**. Characteristics of *PIF6* transcripts and encoded proteins in *Ae. arabicum*.
**Table S3**. Characteristics of *ABI3* transcripts and encoded proteins in *Ae. arabicum*.
**Dataset S1**. List of genes in v3.1 not found or broken in v3.0 and OrthoFinder‐specific genes.
**Dataset S2**. Classification of *Ae. arabicum* MADS MIKC^C^‐type genes and TAP version and Brassicales species comparisons using TAPscan.

## Data Availability

The Illumina and PacBio transcript raw data can be found at the SRA, and annotation and sequence files produced in this article can be found at the *Aethionema arabicum* DB (https://plantcode.online.uni‐marburg.de/aetar_db) and CoGe, in the entry with genome ID 37218 (https://genomevolution.org/coge/GenomeInfo.pl?gid=37218).
