# Supplementary material for: Aethionema arabicum genome annotation using PacBio full‐length transcripts provides a valuable resource for seed dormancy and Brassicaceae evolution research
Source: Plant J. 2021 Feb 8;106(1):275–93. doi: 10.1111/tpj.15161 (PMC8641386; doi:10.1111/tpj.15161)
Supplement: Supplementary file 1 — Figure S1. Repetitive content across Brassicaceae. Figure S2. Alternative splicing in MAKER isoforms. Figure S3. Alternative splicing in PacBio full‐length isoforms. Figure S4. Phylogenetic relationships of the Brassicales species included in OrthoFinder and TAP analysis. Figure S5. Count of transcription associated proteins (TAPs) of Ae. arabicum in comparison with other Brassicales. Figure S6. Phylogeny of Type II MADS‐box genes from Ae. arabicum and other representative flowering plant species. Figure S7. PIF6 alternative splicing isoforms in Ae. arabicum. Figure S8. ABI3 alternative splicing isoforms in Ae. arabicum. Figure S9. ABI4 isoforms and expression in the Ae. arabicum DB genome browser. Figure S10. Strand‐dependent cDNA synthesis and PCR analysis for sense and antisense strands of ABI4 in TUR and CYP. Figure S11. DOG1 alternative splicing isoforms in Ae. arabicum (A) and A. thaliana (B) shown in the Ae. arabicum DB and TAIR genome browsers, respectively. Figure S12. NCED6 isoforms in the Ae. arabicum DB genome browser. Figure S13. Annotation edit distance curves for several training sets for SNAP and Augustus. Table S1. PacBio sequencing statistics. Table S2. Characteristics of PIF6 transcripts and encoded proteins in Ae. arabicum. Table S3. Characteristics of ABI3 transcripts and encoded proteins in Ae. arabicum. Dataset S1. List of genes in v3.1 not found or broken in v3.0 and OrthoFinder‐specific genes. Dataset S2. Classification of Ae. arabicum MADS MIKCC‐type genes and TAP version and Brassicales species comparisons using TAPscan. [file TPJ-106-275-s001.zip › tpj15161-sup-0014-TableS1.docx]

**Table S1. PacBio sequencing statistics.**

| Type^1^ | read number | mean length | Mean quality | mean number of passes | Total base number | Library size fragmentation |
| --- | --- | --- | --- | --- | --- | --- |
| CYP 1 | 48,986 | 1,192 bp | 94% | 17.0 | 58,397,212 | 0.5-1 kbp |
| CYP 2 | 79,515 | 1,478 bp | 96% | 15.4 | 117,557,780 | 1-2 kbp |
| CYP 3 | 82,166 | 1,994 bp | 95% | 11.3 | 163,900,973 | >2 kbp |
| TUR 1 | 52,395 | 1,034 bp | 95% | 21.5 | 54,216,617 | 0.5-1 kbp |
| TUR 2 | 77,833 | 1,399 bp | 96% | 15.4 | 108,953,869 | 1-2 kbp |
| TUR 3 | 77,958 | 1,785 bp | 95% | 13.9 | 139,178,076 | >2 kbp |

^1^Six libraries of cDNA from a mix of RNA from seeds and leaves of the *Ae. arabicum* ecotypes Cyprus (CYP) and Turkey (TUR), were sequenced using the Pacific Biosciences RSII with multiple passes (Iso-seq).
