# Supplementary material for: Aethionema arabicum genome annotation using PacBio full‐length transcripts provides a valuable resource for seed dormancy and Brassicaceae evolution research
Source: Plant J. 2021 Feb 8;106(1):275–93. doi: 10.1111/tpj.15161 (PMC8641386; doi:10.1111/tpj.15161)
Supplement: Supplementary file 1 — Figure S1. Repetitive content across Brassicaceae. Figure S2. Alternative splicing in MAKER isoforms. Figure S3. Alternative splicing in PacBio full‐length isoforms. Figure S4. Phylogenetic relationships of the Brassicales species included in OrthoFinder and TAP analysis. Figure S5. Count of transcription associated proteins (TAPs) of Ae. arabicum in comparison with other Brassicales. Figure S6. Phylogeny of Type II MADS‐box genes from Ae. arabicum and other representative flowering plant species. Figure S7. PIF6 alternative splicing isoforms in Ae. arabicum. Figure S8. ABI3 alternative splicing isoforms in Ae. arabicum. Figure S9. ABI4 isoforms and expression in the Ae. arabicum DB genome browser. Figure S10. Strand‐dependent cDNA synthesis and PCR analysis for sense and antisense strands of ABI4 in TUR and CYP. Figure S11. DOG1 alternative splicing isoforms in Ae. arabicum (A) and A. thaliana (B) shown in the Ae. arabicum DB and TAIR genome browsers, respectively. Figure S12. NCED6 isoforms in the Ae. arabicum DB genome browser. Figure S13. Annotation edit distance curves for several training sets for SNAP and Augustus. Table S1. PacBio sequencing statistics. Table S2. Characteristics of PIF6 transcripts and encoded proteins in Ae. arabicum. Table S3. Characteristics of ABI3 transcripts and encoded proteins in Ae. arabicum. Dataset S1. List of genes in v3.1 not found or broken in v3.0 and OrthoFinder‐specific genes. Dataset S2. Classification of Ae. arabicum MADS MIKCC‐type genes and TAP version and Brassicales species comparisons using TAPscan. [file TPJ-106-275-s001.zip › tpj15161-sup-0019-Supinfo.docx]

***Aethionema arabicum* genome annotation using PacBio full-length transcripts provides a valuable resource for seed dormancy and Brassicaceae evolution research**

**Supplemental materials**


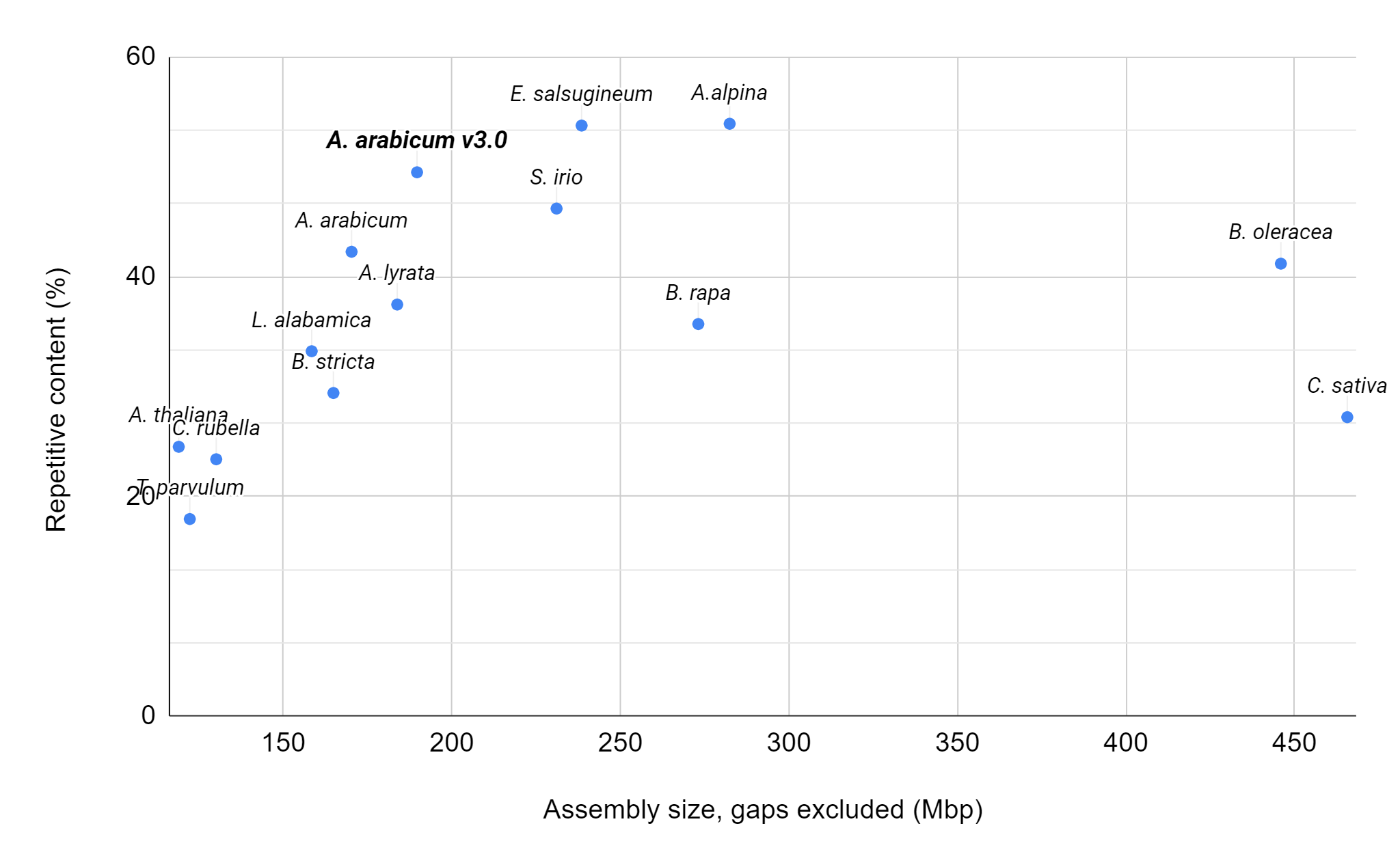


**Figure S1. Repetitive content across Brassicaceae.**

Each point represents the repetitive content as a function of assembly size (gaps excluded) for a given genome assembly.


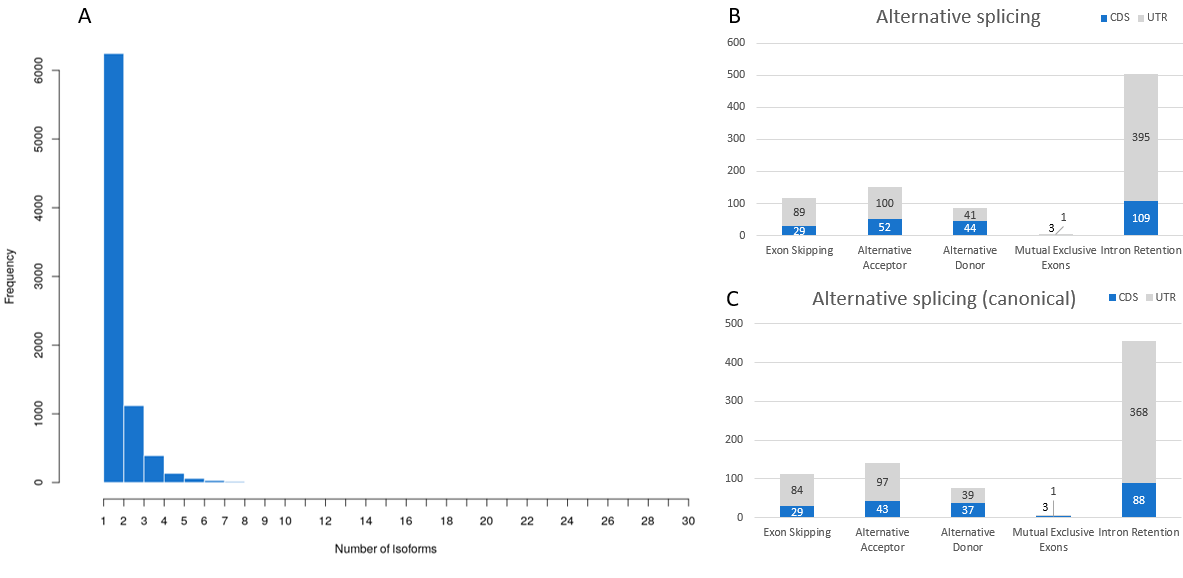


**Figure S2. Alternative splicing in MAKER isoforms.**

**A.** Isoform frequency per gene. **B.** Alternative splicing type count for exon skipping, alternative acceptor, alternative donor, mutually exclusive exons and intron retention events. **C.** Alternative splicing type count for the same events but only when canonical splice sites and basic splicing rules were used.


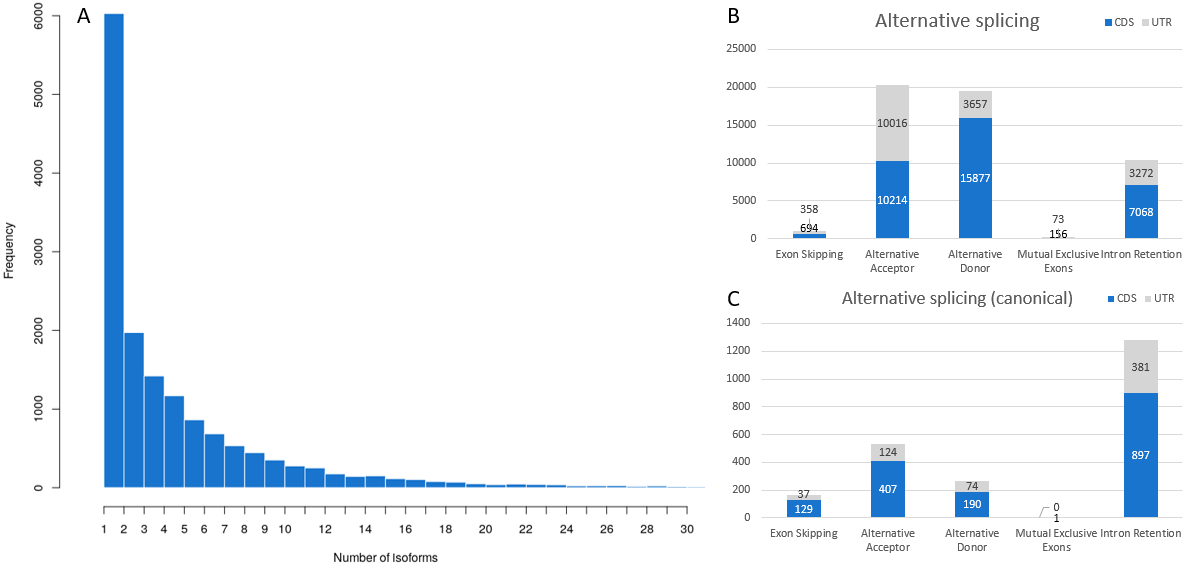


**Figure S3. Alternative splicing in PacBio full-length isoforms.**

**A.** Isoform frequency per gene. **B.** Alternative splicing type count for exon skipping, alternative acceptor, alternative donor, mutually exclusive exons and intron retention events. **C.** Alternative splicing type count for the same events but only when canonical splice sites and basic splicing rules were used.


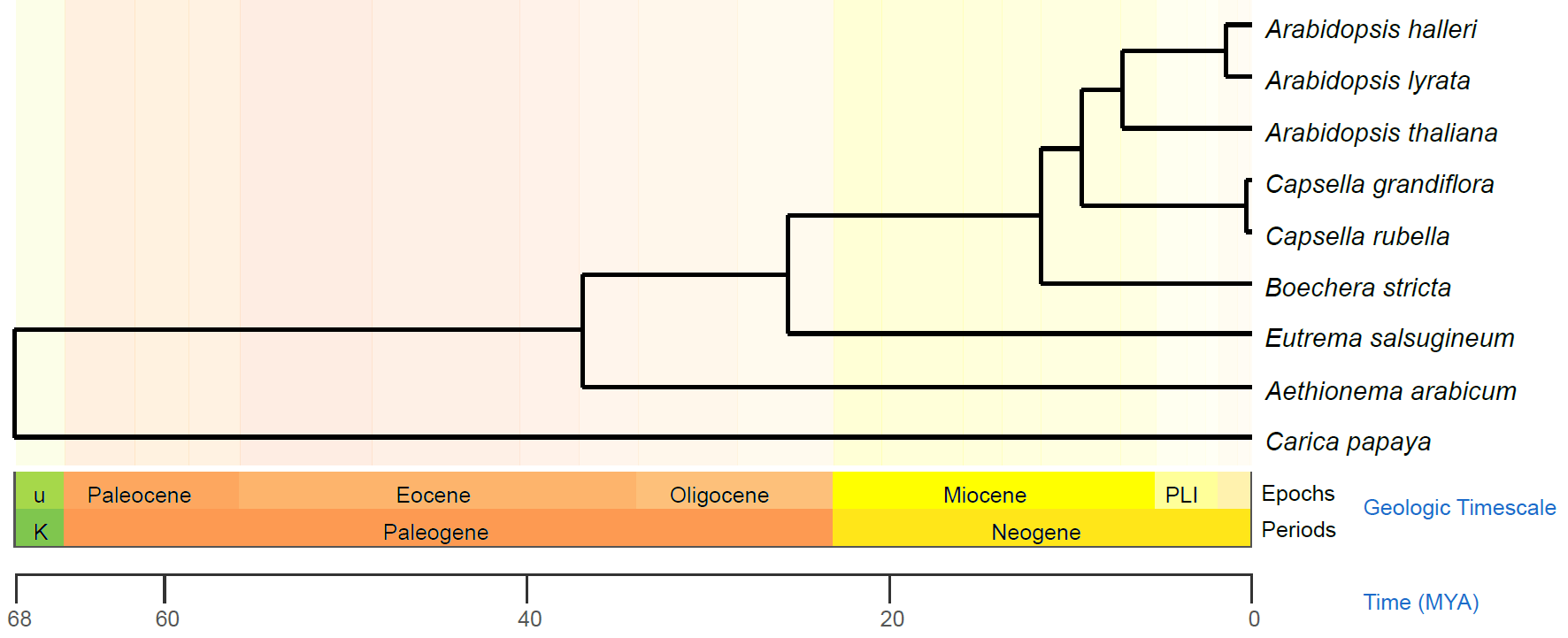


**Figure S4. Phylogenetic relationships of the Brassicales species included in OrthoFinder and TAP analysis.**

Generated in timetree.org and based on (Nikolov et al., 2019).


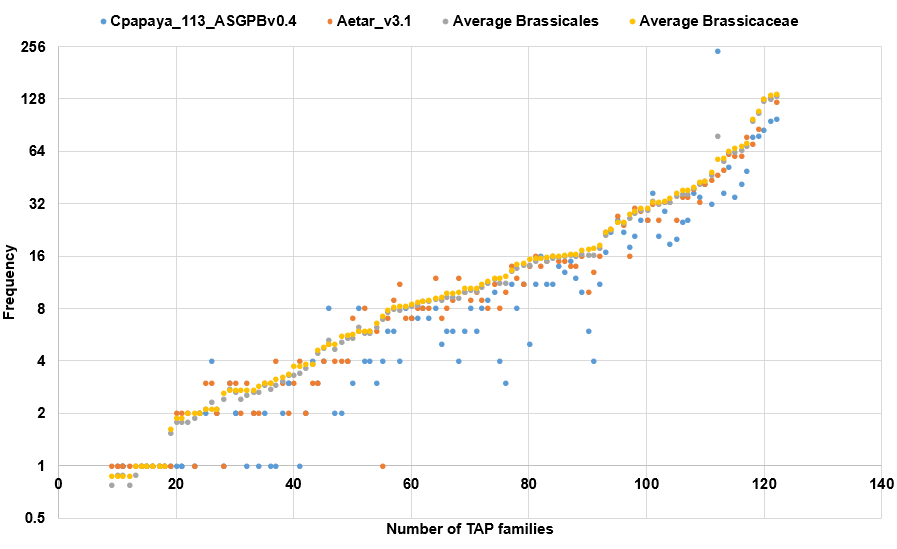


**Figure S5. Count of Transcription Associated Proteins (TAPs) of *Ae. arabicum* in comparison with other Brassicales***.*

*A. thaliana, A. helleri, A. lyrata, C. grandiflora, C. rubella, B. stricta, E. salsugineum* and *Ae. arabicum* are Brassicaceae, while all of them and *C. papaya* are Brassicales. X-axis displays the number of TAP families from the supplemental Data Set 2 sorted by frequency.

**Figure S6.** **Phylogeny of Type II MADS-box genes from Ae. arabicum and other representative flowering plant species.**
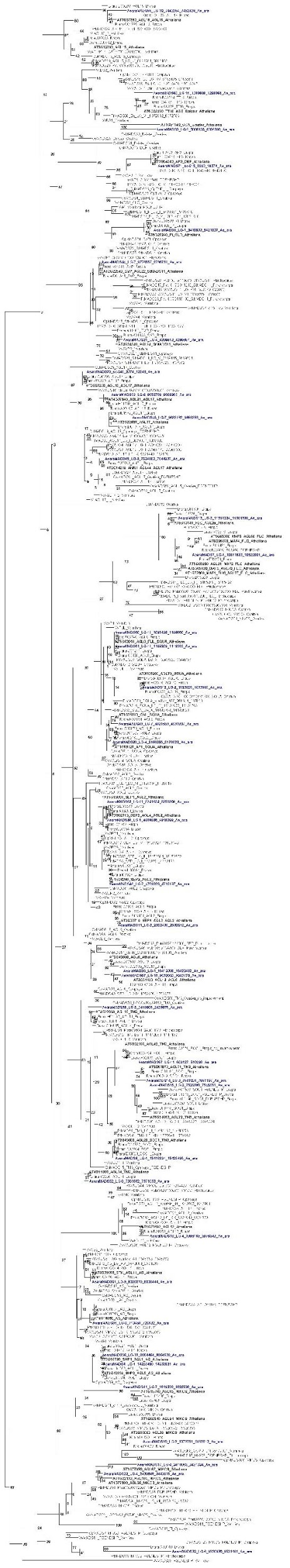


Abbreviations of the species from which the genes where identified are given at the end of the names (Ae_are, *Aethionema arabicum*; Athaliana, *Arabidopsis thaliana*; Brapa, *Brassica rapa*; Cpapaya, *Carica papaya*; Osativa, *Oryza sativa*). Genes from *A. arabicum* and *A. thaliana* are highlighted in bold where genes from *Ae. arabicum* are colored blue. Genes were named according to this paper (*Ae. arabicum*), TAIR (*A. thaliana*), the Brassica database (*B. rapa*), Gramzow and Theißen (2015) (*C. papaya*), (Leseberg et al., 2006) (*P. trichocarpa*) and (Arora et al., 2007) (*O. sativa*). For some genes, the clade to which these genes belong is included in the name (MIKCS, AGL2, FLC, SQUA, etc.).


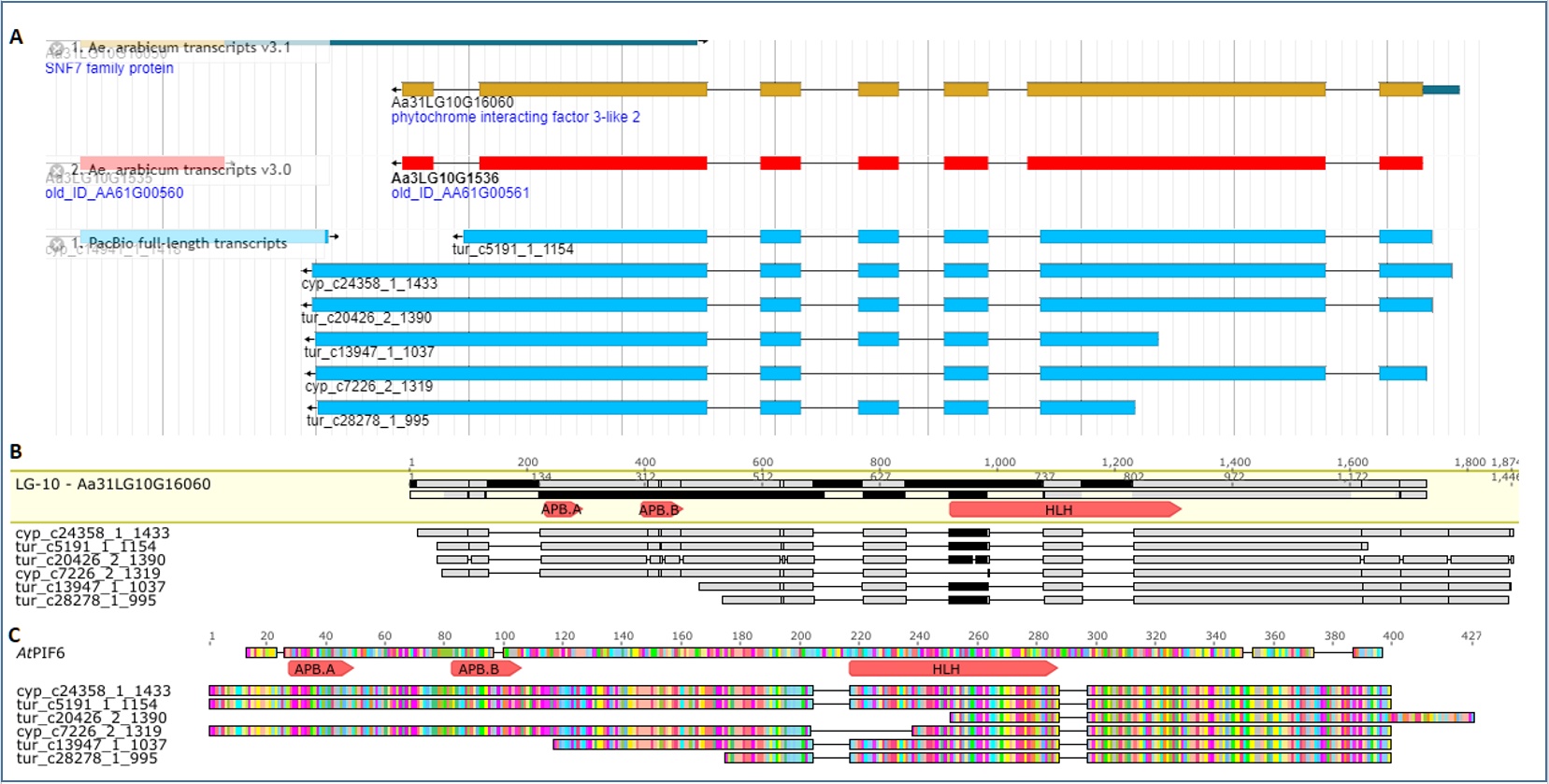


**Figure S7. PIF6 alternative splicing isoforms in *Ae. arabicum*.**

Browser representation of isoforms (A); Annotation of APB and HLH domain-encoding nucleotide sequences aligned to transcript isoforms (B) and annotation of APB and HLH domains aligned to predicted isoforms encoded by transcript ORFs. APB domain annotation based on (Golonka et al., 2019).


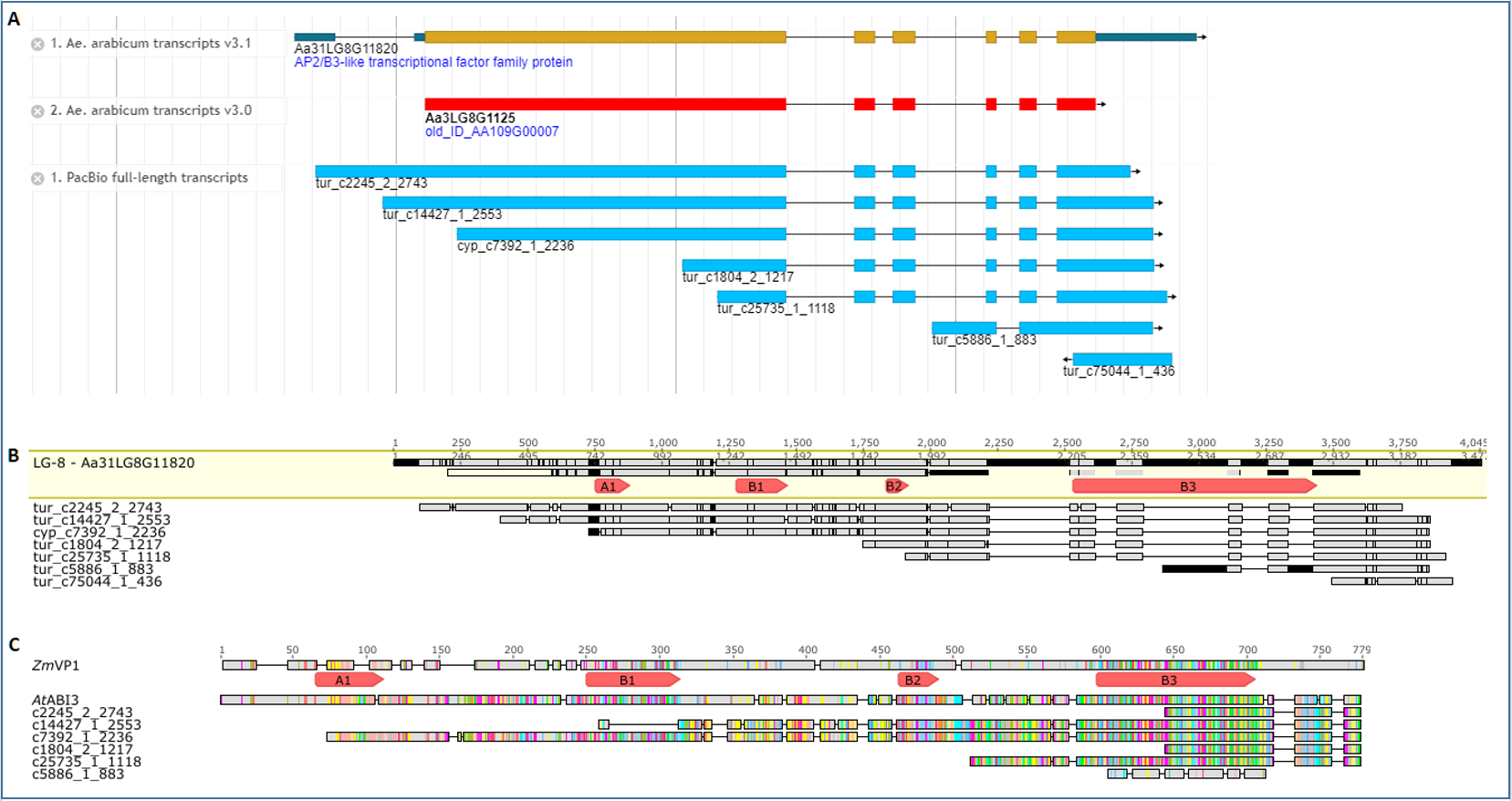


**Figure S8. ABI3 alternative splicing isoforms in *Ae. arabicum.***

Browser representation of isoforms (A); Annotation of A1, B1, B2 and B3 domain-encoding nucleotide sequences aligned to transcript isoforms (B) and annotation of domains aligned to predicted isoforms encoded by transcript ORFs. A1, B1, B2 and B3 domains are annotated based on *Zm*VP1 homology as identified in (Nakamura and Toyama, 2001).


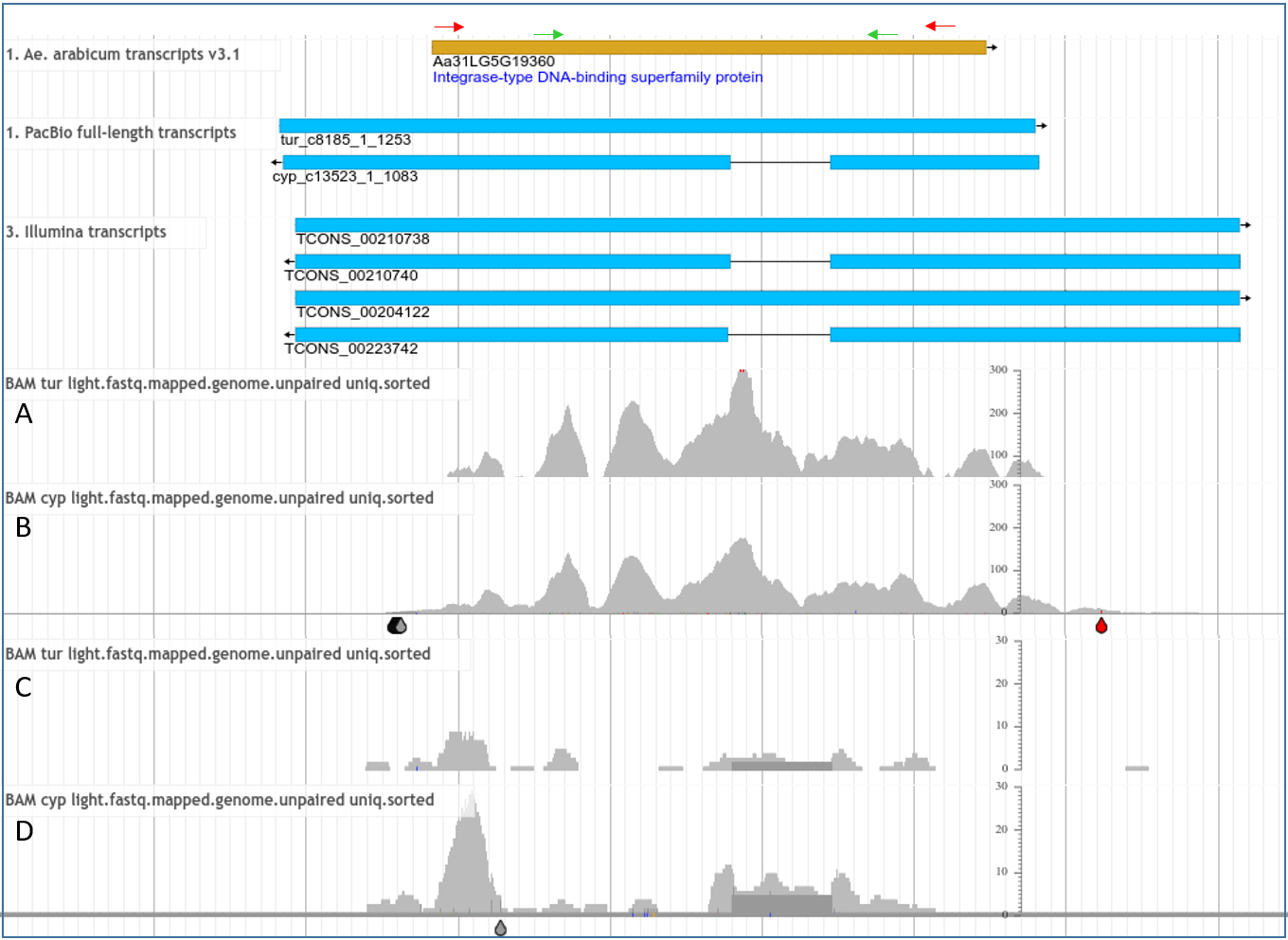


**Figure S9. ABI4 isoforms and expression in the *Ae. arabicum* DB genome browser.**

ABI4 full-length transcripts shows two isoforms, one in TUR in forward and another one in CYP in reverse, both supported by Illumina transcripts. Read depth RNA-seq data from Mérai et al., 2019 are shown below for TUR (A, C) and CYP (B, D) under light conditions in forward (A, B; scale 0 to 300) and reverse (C, D; scale 0 to 30) strands respectively. Color labels under read depth tracks show SNPs of CYP sequences mapped on TUR genome reference. Position of primers used for strand-dependent cDNA synthesis (red arrows) and transcript detection (green arrows) are shown above the ABI4 transcript.


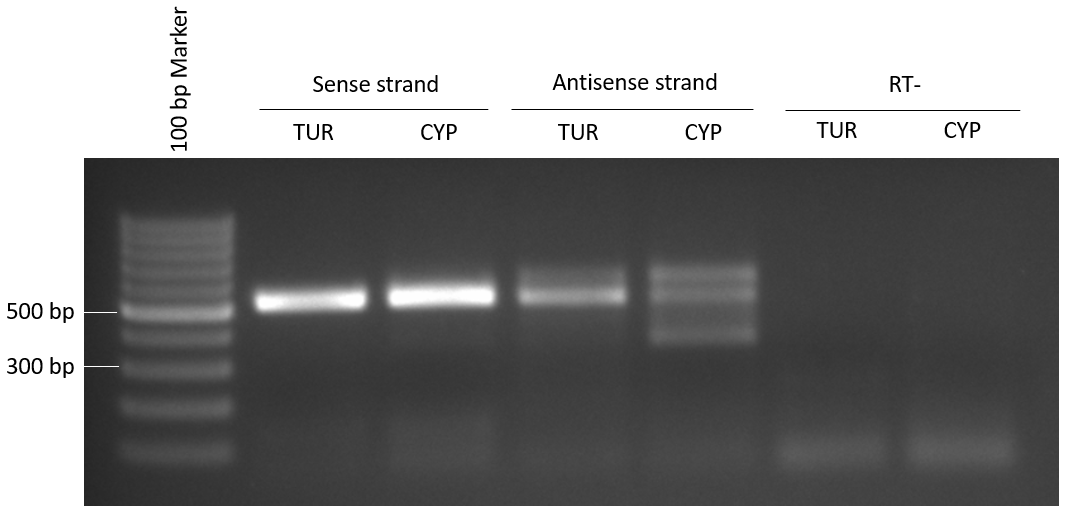


**Figure S10. Strand-dependent cDNA synthesis and PCR analysis for sense and antisense strand of ABI4 in TUR and CYP.**

Sense and antisense transcripts were confirmed for both ecotypes TUR and CYP, the antisense spliced variant was only found in CYP. Size without intron splicing is 533 bp, and after intron splicing is 368 bp.

**
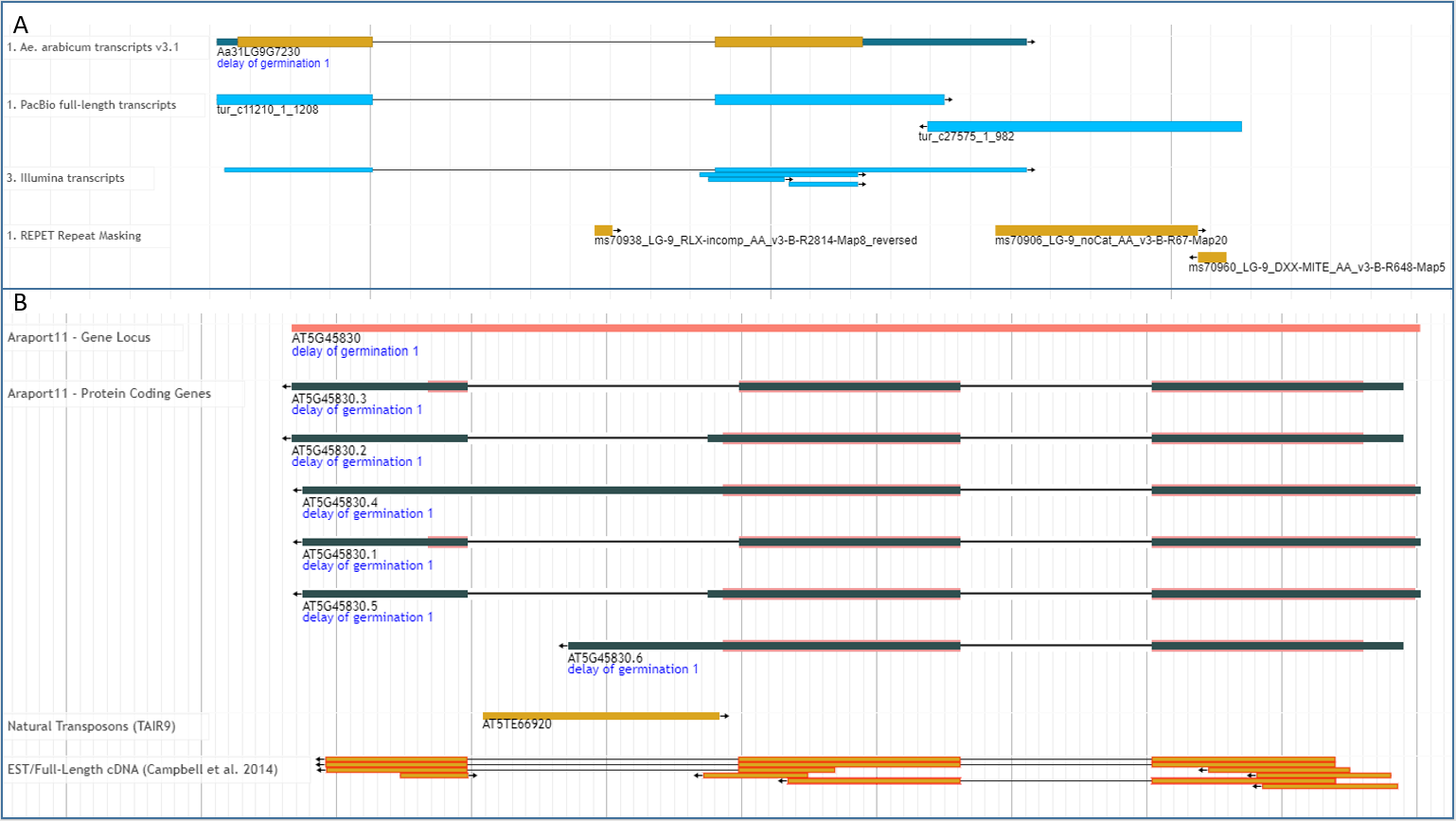
**

**Figure S11. DOG1 alternative splicing isoforms in *Ae. arabicum* (A) and *A. thaliana* (B) shown in the *Ae. arabicum* DB and TAIR genome browsers, respectively.**

aearDOG1 shows only one isoform with 2 exons, supported by the PacBio and Illumina transcripts. In both species, there is an antisense transcript after the second exon and evidences of repeats.


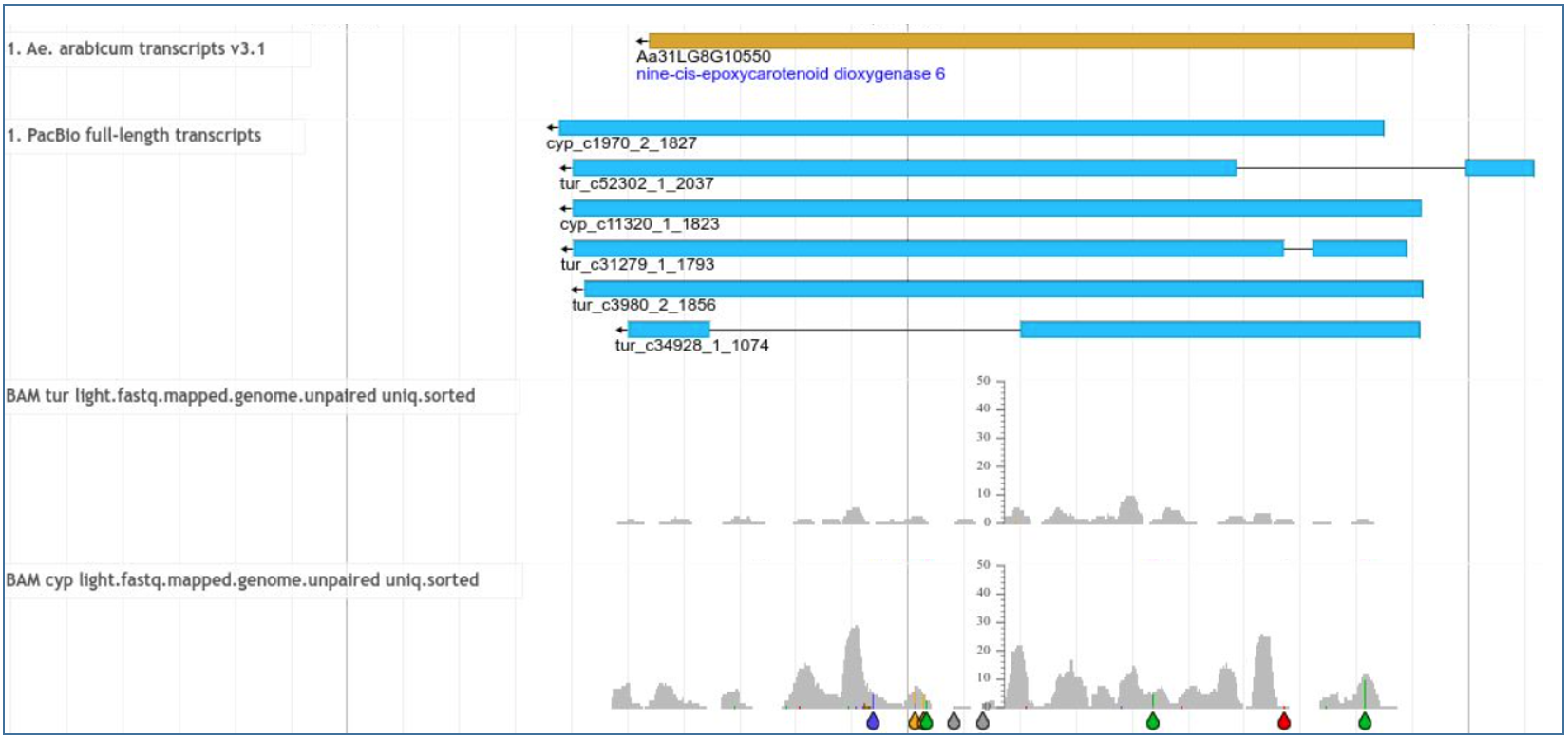


**Figure S12. NCED6 isoforms in the *Ae. arabicum* DB genome browser.**

From top to bottom the next tracks are shown: NCED6 gene model, PacBio full-length transcripts, and RNA-seq mapped read depth for TUR and CYP in light conditions from Mérai et al., 2019, respectively. NCED6 shows six isoforms, two of CYP and four of TUR. Three of the TUR isoforms show introns. Color labels under read depth tracks show SNPs of CYP sequences mapped on TUR genome reference.


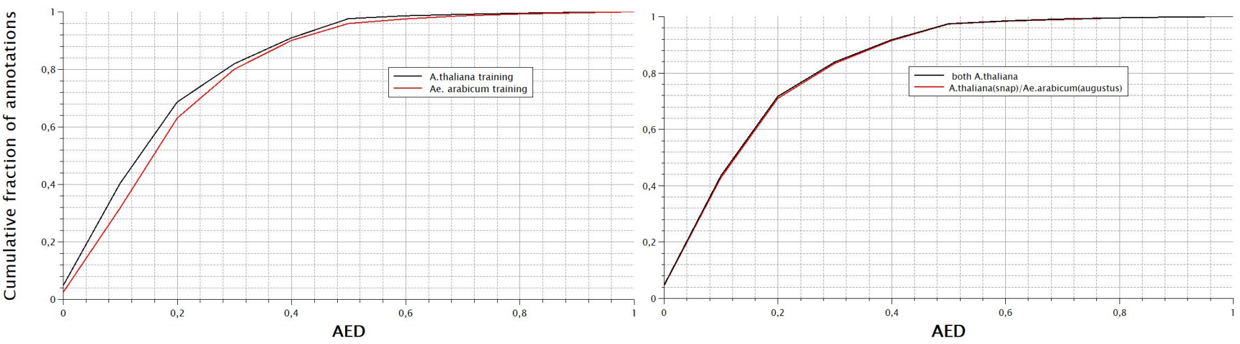


**Figure S13. Annotation Edit Distance curves for several training sets for SNAP and Augustus.**

MAKER results using Trinity transcripts, PacBio full-length transcripts, Embryophyta SwissProt and v3.0 annotation proteins were evaluated using *Ae. arabicum* (red line) and *A. thaliana* (black line) training sets for SNAP (on the left) and Augustus (on the right).

**Table S1. PacBio sequencing statistics.**

| Type^1^ | read number | mean length | Mean quality | mean number of passes | Total base number | Library size fragmentation |
| --- | --- | --- | --- | --- | --- | --- |
| CYP 1 | 48,986 | 1,192 bp | 94% | 17.0 | 58,397,212 | 0.5-1 kbp |
| CYP 2 | 79,515 | 1,478 bp | 96% | 15.4 | 117,557,780 | 1-2 kbp |
| CYP 3 | 82,166 | 1,994 bp | 95% | 11.3 | 163,900,973 | >2 kbp |
| TUR 1 | 52,395 | 1,034 bp | 95% | 21.5 | 54,216,617 | 0.5-1 kbp |
| TUR 2 | 77,833 | 1,399 bp | 96% | 15.4 | 108,953,869 | 1-2 kbp |
| TUR 3 | 77,958 | 1,785 bp | 95% | 13.9 | 139,178,076 | >2 kbp |

^1^Six libraries of cDNA from a mix of RNA from seeds and leaves of the *Ae. arabicum* ecotypes Cyprus (CYP) and Turkey (TUR), were sequenced using the Pacific Biosciences RSII with multiple passes (Iso-seq).

**Table S2. Characteristics of *PIF6* transcripts and encoded proteins in *Ae. arabicum.***

**
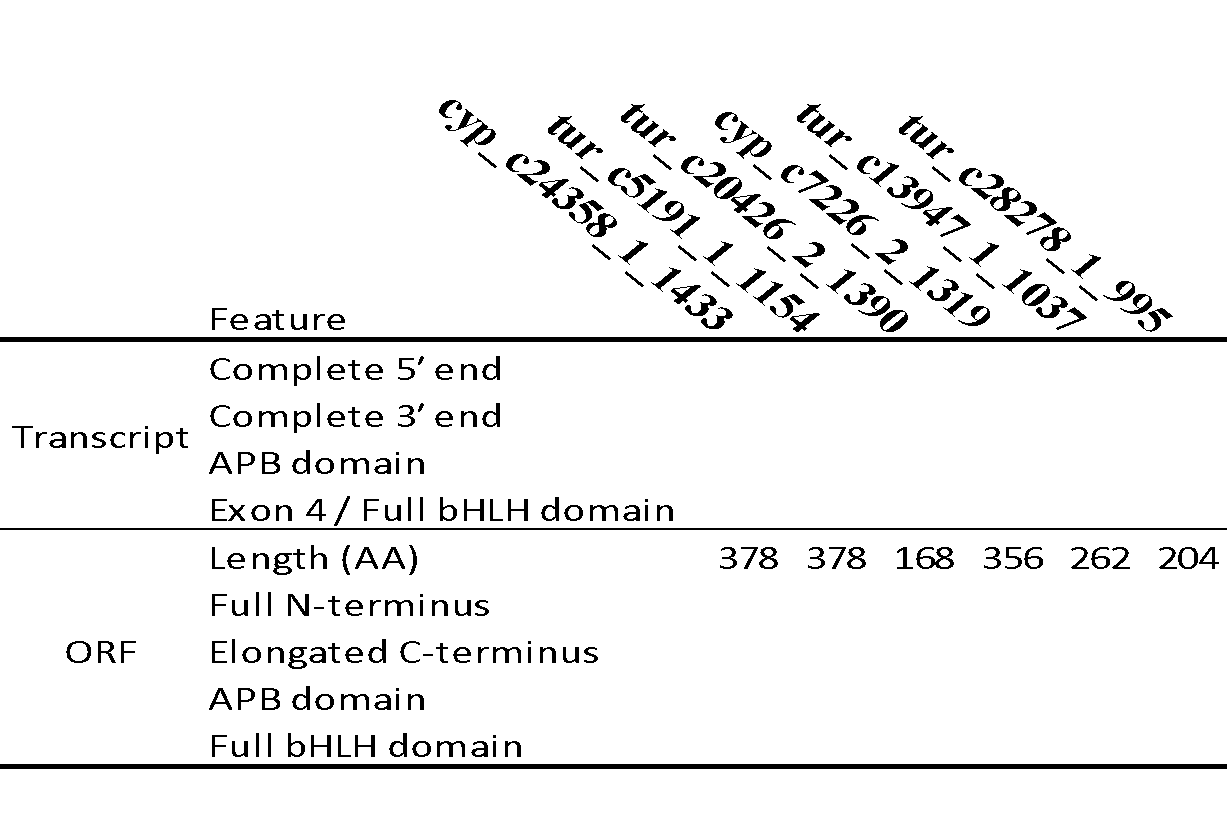
**

**Table S3. Characteristics of *ABI3* transcripts and encoded proteins in *Ae. arabicum.***


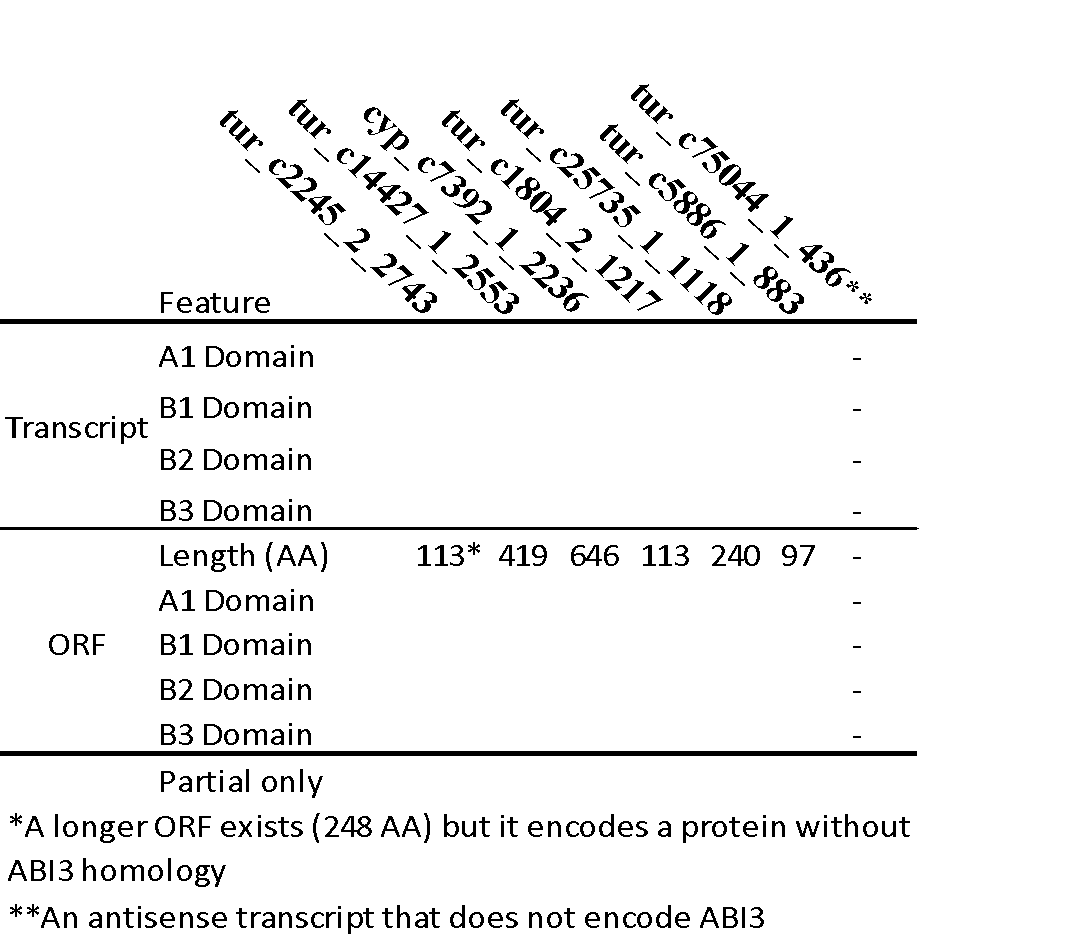


**References**

**Arora, R., Agarwal, P., Ray, S., Singh, A.K., Singh, V.P., Tyagi, A.K., and Kapoor, S.** (2007). MADS-box gene family in rice: genome-wide identification, organization and expression profiling during reproductive development and stress. BMC Genomics **8,** 242.

**Golonka, D., Fischbach, P., Jena, S.G., Kleeberg, J.R.W., Essen, L.O., Toettcher, J.E., Zurbriggen, M.D., and Moglich, A.** (2019). Deconstructing and repurposing the light-regulated interplay between Arabidopsis phytochromes and interacting factors. Communications Biology **2**.

**Leseberg, C.H., Li, A., Kang, H., Duvall, M., and Mao, L.** (2006). Genome-wide analysis of the MADS-box gene family in Populus trichocarpa. Gene **378,** 84-94.

**Nakamura, S., and Toyama, T.** (2001). Isolation of a VP1 homologue from wheat and analysis of its expression in embryos of dormant and non-dormant cultivars. Journal of Experimental Botany **52,** 875-876.

**Nikolov, L.A., Shushkov, P., Nevado, B., Gan, X., Al-Shehbaz, I.A., Filatov, D., Bailey, C.D., and Tsiantis, M.** (2019). Resolving the backbone of the Brassicaceae phylogeny for investigating trait diversity. New Phytol **222,** 1638-1651.
